# Biocompatible Self-Assembled Hydrogen-Bonded Gels Based on Natural Deep Eutectic Solvents and Hydroxypropyl Cellulose with Strong Antimicrobial Activity

**DOI:** 10.3390/gels8100666

**Published:** 2022-10-17

**Authors:** Daniela Filip, Doina Macocinschi, Mihaela Balan-Porcarasu, Cristian-Dragos Varganici, Raluca-Petronela Dumitriu, Dragos Peptanariu, Cristina Gabriela Tuchilus, Mirela-Fernanda Zaltariov

**Affiliations:** 1 Laboratory of Physical Chemistry of Polymers, “Petru Poni” Institute of Macromolecular Chemistry, Aleea Gr. Ghica Voda 41 A, 700487 Iasi, Romania; 2 Laboratory of Polycondensation and Thermostable Polymers, “Petru Poni” Institute of Macromolecular Chemistry, Aleea Gr. Ghica Voda 41 A, 700487 Iasi, Romania; 3 Centre of Advanced Research in Bionanoconjugates and Biopolymers, “Petru Poni” Institute of Macromolecular Chemistry, Aleea Gr. Ghica Voda 41 A, 700487 Iasi, Romania; 4 Microbiology Department, Faculty of Medicine, “Gr. T. Popa” University of Medicine and Pharmacy, 16 Universitatii Street, 700115 Iasi, Romania; 5 Department of Inorganic Polymers, “Petru Poni” Institute of Macromolecular Chemistry, Aleea Gr. Ghica Voda 41 A, 700487 Iasi, Romania

**Keywords:** deep eutectic solvents, hydroxypropyl cellulose, rheology, antimicrobial gels, biocompatibility

## Abstract

Natural deep eutectic solvents (NADES)-hydroxypropyl cellulose (HPC) self-assembled gels with potential for pharmaceutical applications are prepared. FT-IR, ^1^HNMR, DSC, TGA and rheology measurements revealed that hydrogen bond acceptor–hydrogen bond donor interactions, concentration of NADES and the water content influence significantly the physico-chemical characteristics of the studied gel systems. HPC-NADES gel compositions have thermal stabilities lower than HPC and higher than NADES components. Thermal transitions reveal multiple glass transitions characteristic of phase separated systems. Flow curves evidence shear thinning (pseudoplastic) behavior. The flow curve shear stress vs. shear rate were assessed by applying Bingham, Herschel–Bulkley, Vocadlo and Casson rheological models. The proposed correlations are in good agreement with experimental data. The studied gels evidence thermothickening behavior due to characteristic LCST (lower critical solution temperature) behavior of HPC in aqueous systems and a good biocompatibility with normal cells (human gingival fibroblasts). The order of antibacterial and antifungal activities (*S.aureus*, *E.coli*, *P. aeruginosa* and *C. albicans*) is as follows: citric acid >lactic acid > urea > glycerol, revealing the higher antibacterial and antifungal activities of acids.

## 1. Introduction

Natural deep eutectic solvents (NADES) belong to a new generation of solvents [1,2,3] comprising mixtures of cheap and readily obtainable components which generate eutectics with melting points significantly lower that of their individual components due to ion–dipole interaction or hydrogen bonding. They are ionic green solvents which are not volatile, not flammable and a low-cost alternative to room temperature ionic liquids. Their use opens the possibility of replacing toxic imidazolium ionic liquids by more sustainable compounds that can be applied in the development of biocompatible and biodegradable drug delivery responsive systems [4].

Their unique properties recommend them in varied practical applications ranging from extraction and biocatalysis to biomedical ones. In biomedical field NADES can be used as biopolymer modifiers, acting as template delivery compounds also knows as “therapeutic deep eutectic solvents”, being able to solubilize and stabilize different pharmaceutical products [5].

NADES are interesting liquid-like gels in which H-bonds mediate anion binding, and are able to produce major changes in bulk material characteristics. The field of “eutectogels” is less developed than that of ionogels based on classical ionic liquids. Thus, NADES can be used as template for supramolecular gelators [6], being environmentally friendly and providing low preparation costs, non-inflammability, increased thermal and chemical stability, low toxicity and biodegradability. They are developed as a good alternative for poor soluble drugs, being used as excipients in the pharmaceutical industry [7].

In NADES, choline chloride is the hydrogen bond acceptor (HBA) and the other natural components such as alcohols, acids, amides, amines and sugars are hydrogenbond donors (HBD). Choline chloride is a substituted quaternary ammonium salt, a human and animal nutrient [8]. It is found in high quantities in eggs, liver, peanuts, meats and vegetables. This advantage promises great possibilities for drug-delivery systems, bone-therapy scaffolds, cosmetic, pharmaceutical and food-related applications [9]. It was suggested that NADES represent the third liquid phase in organisms [2]. Their increased viscosities constitute a disadvantage but adding water is employed to modulate physicochemical properties and facilitate their applications [10].

Water-based NADES can be an alternative to DESs of high viscosities, poor conductivities and higher toxicities [11]. The organic solvents are not adequate for pharmaceutical applications, therefore NADES are preferred to solubilize the hydrophobic drugs. Interest in NADES for pharmaceutical applications is recent [12,13]. They possess intrinsic antimicrobial activity, can promote absorption and diminish phenomena like polymorphism or degradation.

Hydroxypropyl cellulose (HPC) is a cellulose ether derivative which is obtained by hydroxypropylation of hydroxyl groups of the cellulose backbone, i.e., by reacting alkali cellulose with propylene oxide at elevated pressure and temperature. The hydroxyl groups as well as the incorporated hydroxypropyl groups are able to donate hydrogen bonds to active pharmaceutical ingredients with hydrogen accepting groups [14]. HPC serves well as an excipient for pharmaceuticals. It is also used as emulsifier, stabilizer and thickener in pharmaceutical applications, additives in cosmetics, formulation aid and texturizer for foods.

In this study, we investigate the properties of hydrophilic polymer matrices comprising HPC and four different NADES (NADES gels): choline chloride: urea 1:2 (ChCl-U), choline chloride: citric acid 1:1 (ChCl-CA), choline chloride: lactic acid 1:1 (ChCl-LA), choline chloride: glycerol 1:2 (ChCl-Gly) in aqueous systems. The rheological behavior of HPC gels at various concentrations of NADES is investigated. The thermal transitions and thermal stability of HPC-NADES compositions are investigated by DSC and TGA analyses, respectively. The strength of hydrogen bonding in the HPC-NADES gels is investigated by means of FT-IR and ^1^HNMR spectroscopy. The antimicrobial activities of pure NADES and NADES-HPC gels were studied against Gram positive bacteria (*Staphylococcus aureus* ATCC 25923), Gram negative bacteria (*Escherichia coli* ATCC 25922, *Pseudomonas aeruginosa* ATCC 27853) and pathogenic yeasts (*Candida albicans* ATCC 90028). The biocompatibility of NADES gels was evaluated on HGF (human gingival fibroblast) cell line.

## 2. Results and Discussion

### 2.1. FT-IR Spectroscopy

**Figure 1:**
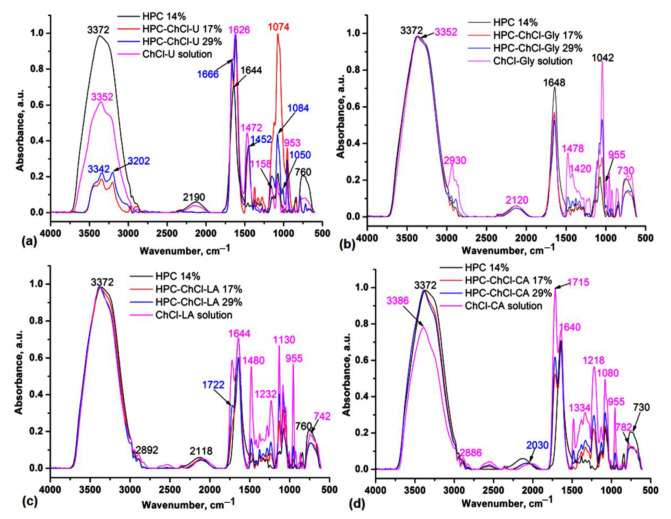
Comparative IR spectra in ATR (Attenuated Total Reflectance) mode of the HPC-ChCl-U (**a**), HPC-ChCl-Gly (**b**), HPC-ChCl-LA (**c**) and HPC-ChCl-CA (**d**) at 17% and 29% NADES concentrations compared with the starting components HPC solution 14% and original NADES in solution.

The HPC-NADES gels are prepared by physical interactions, mainly H-bonding starting from original NADES containing two components: choline chloride (ChCl) as hydrogen acceptor and four different hydrogen donors: urea, glycerol, lactic acid and citric acid. Based on the IR and NMR data it was possible to establish the HPC spectral changes by addition of various amounts of NADES: 17% and 29%, mainly in the O-H spectral region by involving of O-H groups in the H-bonding with NADES components. The IR comparative spectra of HPC solution 14%, original NADES in solution and two HPC-NADES mixtures with 17% and 29% NADES containing water as third component can be seen in Figure 1.

It is evident that the –OH stretching bands of the HPC-NADES aq. solutions are blueshifted by 30 cm^−1^ as compared with HPC solution 14% and by 10 cm^−1^ as compared with original NADES (ChCl-U) in solution (Figure 1a). In the HPC-ChCl-Gly, HPC-ChCl-LA and HPC-ChCl-CA mixtures the O-H stretches are close to those found in HPC solution 14% (Figure 1b–d). These are redshifted by 20 cm^−1^ compared to the corresponding original NADES in solution and confirm their involvement in the H-bonding interactions. The C-H stretches are present at 2962–2886 cm^−1^, while the broad bands at 2540–2030 cm^−1^ are characteristic for H-bonding O-H groups. In the 1800–1500 cm^−1^ spectral range specific stretching vibrations for carboxylic C=O groups in HPC-ChCl-LA and HPC-ChCl-CA can be seen at 1722 and 1715 cm^−1^, respectively (Figure 1c,d).

The C=O stretching band of aq. HPC 14% located at 1682 (sh) and 1644 cm^−1^ is explained with deformative water molecules [15]. The C=O stretching band of HPC-NADES aq. solutions are close to that of HPC solution 14% and influenced by C=O stretching bands of studied NADES. The characteristic bands associated with ChCl located at about 1480 cm^−1^ attributed to ρCH_3_ and the band at 955 cm^−1^ attributed to ammonium structure identity of the NADES (Ch)^+^ are distinct from the bands of HPC aq. solution and decrease their intensities with decreasing concentration of NADES [16]. The C-O-C stretching bands of HPC-NADES aq. solutions located in the 1200–1000 cm^−1^ region are close to those of HPC 14% *w*/*v* and influenced by the bands of NADES.

NADES have similar bands to the starting HBD and HBA molecules [17,18]. For ChCl-CA 17% and ChCl-Gly 17% samples the peaks at 955 cm^−1^ vanish (Figure 1b,d). This result is explained by the strength of hydrogen bonding and less phase separation in comparison with ChCl-LA and ChCl-U NADES. In general, ChCl-U, ChCl-Gly, ChCl-LA and ChCl-CA are high viscosity liquid systems, with high hygroscopicity mainly due to the ChCl component, so that these can be isolated with lower water (adsorbed moisture from surrounding atmosphere) content in their composition. In binary mixtures of studied NADES there is a clear contribution of HBD and HBA role in the system, while in the ternary and quaternary systems, resulting from the addition of water and HPC, there is a more complex hydrogen bonding network in the system.

**Figure 2:**
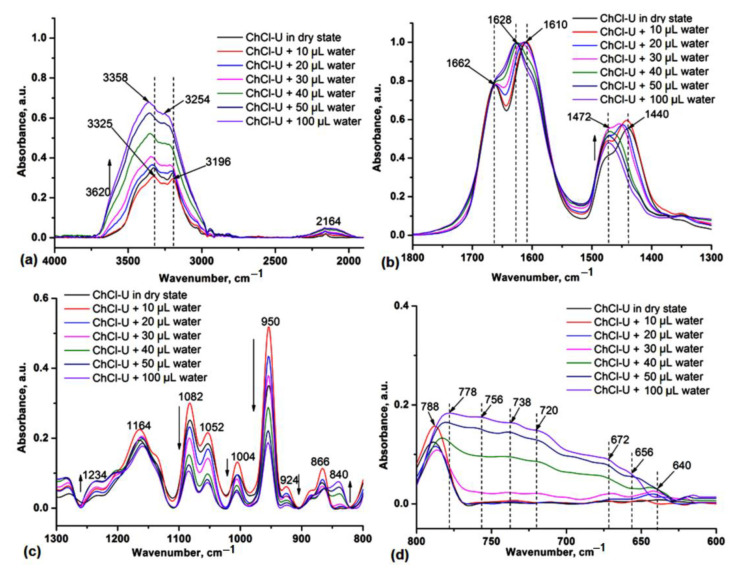
IR spectra of ChCl-U during the addition of water in the 4000–1800 cm^−1^ (**a**), 1800–1300 cm^−1^ (**b**), 1300–800 cm^−1^ (**c**) and 800–600 cm^−1^ (**d**) spectra range. The vertical dashed lines highlight the shifts of the main absorptions in the presence of different amounts of water.

We studied the effect of water addition in the original NADES (prepared in solid state by simple plastering the components) by IR spectroscopy in order to highlight that water can stabilize HBD components in the liquid phase and mediate the interaction with choline chloride (HBA component). In Figure 2 the IR spectra of ChCl-U during the addition of different amounts of water are shown. One can observe the redshift of the O-H and N-H stretches by 33–58 cm^−1^ (Figure 2a), respectively, and of O-H and N-H deformations (Figure 2b) at 1662 and 1610 cm^−1^ by adding water as a third component in the NADES system. These spectral changes confirmed that the strong H-bond interactions between ChCl and urea gradually decrease in the presence of water, also leading to the decrease in the intensity of the characteristic vibrations at 1480 and 955 cm^−1^ (Figure 2c) and the appearance of broad bands at 800–600 cm^−1^, specifically for different H-bonding water molecules (Figure 2d).

**Scheme 1:**
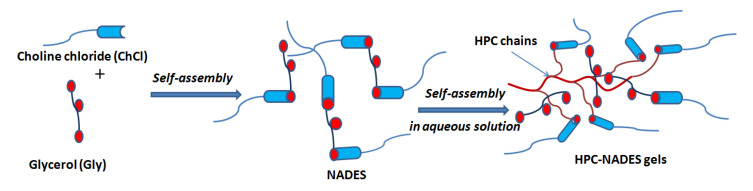
Schematic representation of the reorganization of H-bonding (donor–acceptor) by addition of HPC.

For all HPC-based NADES and water/NADES systems, there was a decrease in H-bond energy by addition of both, water and HPC solution 14%, as compared with these systems in dry state (Table 1). This process is accompanied by the reorganization of the H bonding interactions between both components of NADES, as can be seen in a schematic representation (Scheme 1), by involving water and HPC chains.

The H-bond distance also highlighted this aspect, by slightly increasing values of its value, supporting the interconnectivity between NADES, HPC and/or water components. Moreover, one can see from Table 1 that the H bonds distance of HPC-NADES gels differ slightly in solution and in solid state as compared with original NADES. Higher differences appear to initial HPC, the values of H-bond distance being larger than the HPC in solution. This suggests that original H-bonding NADES co-exist with H-bonded HPC-NADES in the gel mixture. Thus, it was found that the presence of HBD (hydrogen bond donor) molecules favors the interaction with polymer and diminishes the interaction with water molecules and therefore the solute–solvent interactions in the ternary solutions are similar to those in binary solutions. The NADES complex separated into its precursors can be treated as a pseudo-compound when the HBA (hydrogen bond acceptor): HBD molar ratio is maintained and the system is pseudo-ternary. Stronger hydrogen bonding of NADES determines favorable interactions and increased solvation capability of the carbohydrates.

H-bonds energy (E_H_) and distance (R) of original NADES and HPC-NADES solutions (Table 1) have been estimated by using Sederholm and Struszczyk methods by a previously published procedure [19].

One can observe that in dry state in the original NADES there is a strong interaction between both components, the higher energy of the H bonds being found for ChCl-Gly, followed by ChCl-U, ChCl-LA and ChCl-CA, while in water these interactions considerably decrease. For HPC-NADES mixture in dry state, the energy of the H bonds increases at lower concentration (17%) of NADES. A strong interaction was observed for HPC-ChCl-U 29%, which is maintained inclusively in solution of 14% HPC. A lower interaction was found for HPC-ChCl-CA both in dry state and in solution. The value of the distance of H bonds is lower for HPC-ChCl-U and HPC-ChCl-Gly at both concentrations, also proving a strong interaction within the ternary systems.

### 2.2. ^1^H NMR and ROESY Spectra

There are several NMR studies concerning the molecular interactions in NADES based on ChCl and CA, LA, U and Gly, respectively, and their mixtures with water [20,21,22,23,24]. These studies show that adding more than 10% water to the NADES leads to weakening of the hydrogen bond network which is reflected in the ROESY spectra by the disappearance of the intermolecular correlation peaks and in the ^1^H NMR spectra by the collapse of all the peaks for labile protons and water into one peak, due to the fast exchange that takes place in presence of water.

The same trend was observed in the case of our mixtures, where the ROESY spectra (Figures S1–S8) recorded for mixtures of ChCl-U-H_2_O (1:2:6 molar ratio) (Figures S1 and S2), ChCl-Gly-H_2_O (1:2:11 molar ratio) (Figures S3 and S4), ChCl-LA-H_2_O (1:1:7 molar ratio) (Figures S5 and S6) and ChCl-CA-H_2_O (1:1:10 molar ratio) (Figures S7 and S8), and show only intramolecular correlation peaks and no intermolecular interactions.

**Figure 3:**
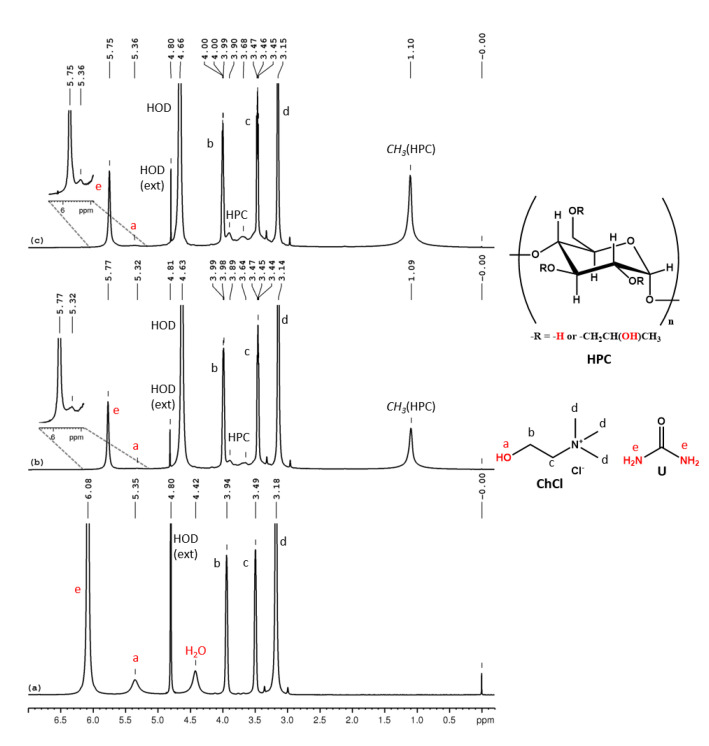
^1^H NMR spectra of ChCl-U 100% (**a**), HPC-ChCl-U 29% (**b**) and HPC-ChCl-U 17% (**c**). In red color are marked the exchangeable protons.

In the ^1^H NMR spectrum for ChCl-U 100% (Figure 3a) we can observe the following peaks (δ, ppm): 6.08 (U-*NH*_2_), 5.35 (ChCl-*OH*), 4.42 (H_2_O), 3.94 (ChCl-*CH*_2_-OH), 3.49 (ChCl-*CH*_2_-N), 3.18 (ChCl-N-(*CH*_3_)_3_). This eutectic mixture also contains intrinsic water (adsorbed water during the preparation) and the molar ratio between ChCl:U:H_2_O, as calculated from the integral ratio, is 1:2:0.6. In the ^1^H NMR spectra of HPC-ChCl-U 29% and HPC-ChCl-U 17% from Figure 3b,c, we can observe the peaks from HPC (1.1 ppm, 3–4.5 ppm, overlapped with the peaks from ChCl) and the peaks for U-*NH*_2_ and ChCl-*OH* are still observed at 5.75–5.77 ppm and 5.32–5.36 ppm, respectively, but their intensity is diminished due to the exchange with deuterium from the solvent. Urea is known to form very strong hydrogen bonds which leads to a slower exchange rate, thus the peaks for the labile protons are still observed in the ^1^H NMR spectra even at higher water contents. A ROESY experiment (Figure S9) was performed for HPC-ChCl-U 17% in order to evaluate if the supramolecular structure of ChCl-U is maintained after mixing it with HPC and water. No intermolecular correlation peaks were observed between the three components, meaning that the hydrogen bond network considerably weakened.

**Figure 4:**
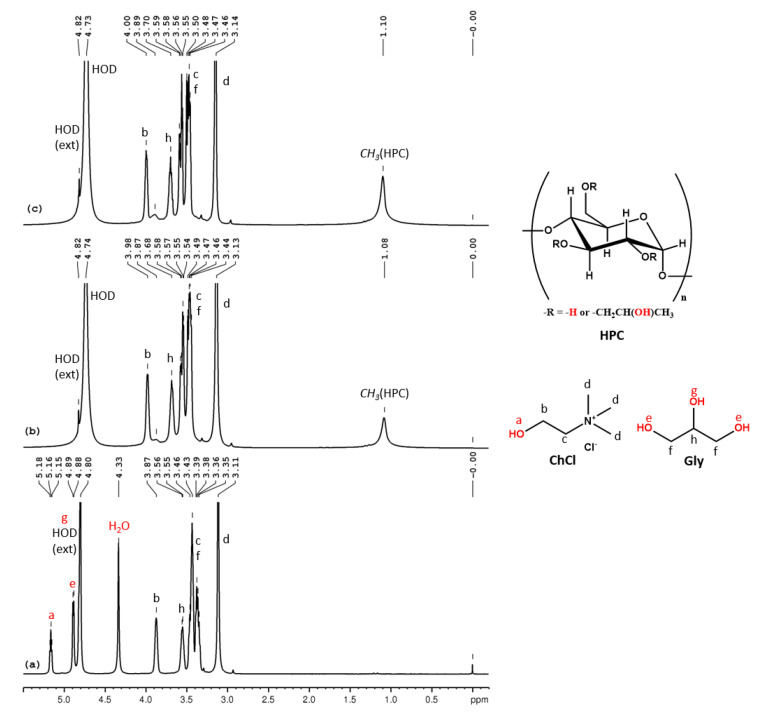
^1^H NMR spectra of ChCl-Gly (**a**), HPC-ChCl-Gly 29% (**b**) and HPC-ChCl-Gly 17% (**c**). In red color are marked the exchangeable protons.

In the ^1^H NMR spectrum of ChCl-Gly 100% (Figure 4a) we can observe the following peaks (δ, ppm): 5.16 (ChCl-*OH)*,4.89 (Gly-CH-*OH*), 4.80 (Gly-CH_2_-*OH*, partially overlapped with external HOD), 4.33 (H_2_O), 3.87 (ChCl-*CH*_2_-OH), 3.55 (ChCl-*CH*_2_-N), 3.46–3.35 (Cly-*CH*_2_- and Gly-*CH*-) and 3.11 (ChCl-N-(*CH*_3_)_3_). Although no water was added during the preparation of ChCl-Gly, the ^1^H NMR spectrum shows the presence of a small content of water. The molar ratio of the ChCl:Gly:H_2_O ternary mixture, calculated from the integral values, is 1:2:2.5. Figure 4b,c shows the ^1^H NMR spectra for two mixtures of different proportions of ChCl-Gly, HPC and D_2_O. We can observe the peak for the methyl group from the 2-hydroxypropyl substituent of cellulose at 1.10 ppm but the rest of the peaks of HPC, which resonate between 3 and 4.5 ppm, are overlapped with the peaks from ChCl and Gly. The hydroxylic protons from ChCl and Gly appear with the HOD peak due to fast exchange with the water protons.

**Figure 5:**
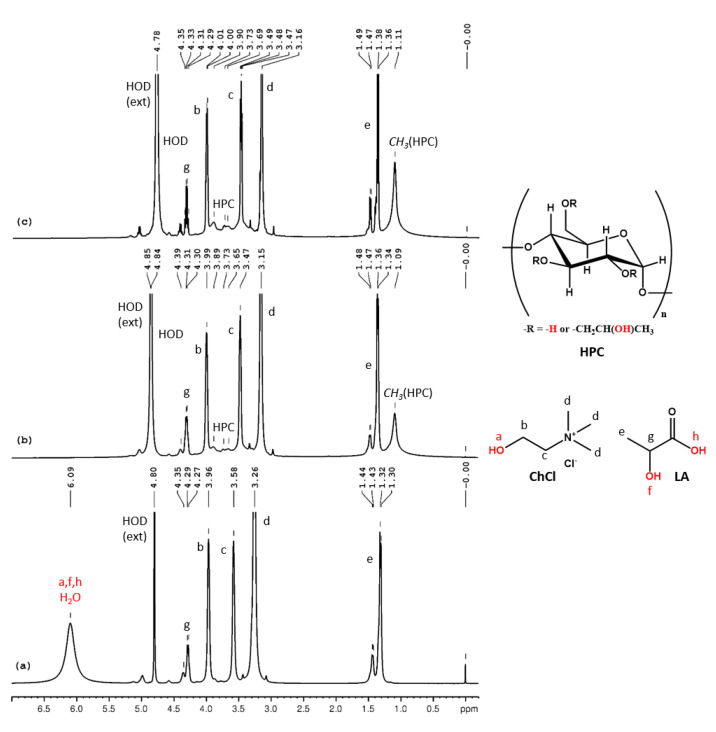
^1^H NMR spectra of ChCl-LA (**a**), HPC-ChCl-LA 29% (**b**) and HPC-ChCl-LA 17% (**c**). In red color are marked the exchangeable protons.

The assignments of the peaks from the ^1^H NMR spectrum of neat ChCl:LA (Figure 5a) are as follows (δ, ppm): 6.09 (LA-*OH*, LA-*COOH*, ChCl-*OH*, H_2_O), 4.28 (LA-*CH*-), 3.96 (ChCl-*CH*_2_-OH), 3.58 (ChCl-*CH*_2_-N), 3.26 (ChCl-N-(*CH*_3_)_3_) and 1.31 (LA-*CH*_3_). Since carboxylic protons are more acidic than alcohols, a faster exchange occurs in the case of ChCl-LA and a single, time-averaged signal for all the labile protons and intrinsic water is observed in the ^1^H NMR spectrum. In Figure 5b,c the ^1^H NMR spectra for HPC-ChCl-U 17% and HPC-ChCl-U 29% are depicted. Since the addition of water increases the rate of exchange, we can observe the displacement of the peak for the labile and water protons from 6.09 ppm to 4.8 ppm and the presence of the HPC peaks (1.10 ppm and 3–4.5 ppm, overlapped with the ChCl and LA peaks).

**Figure 6:**
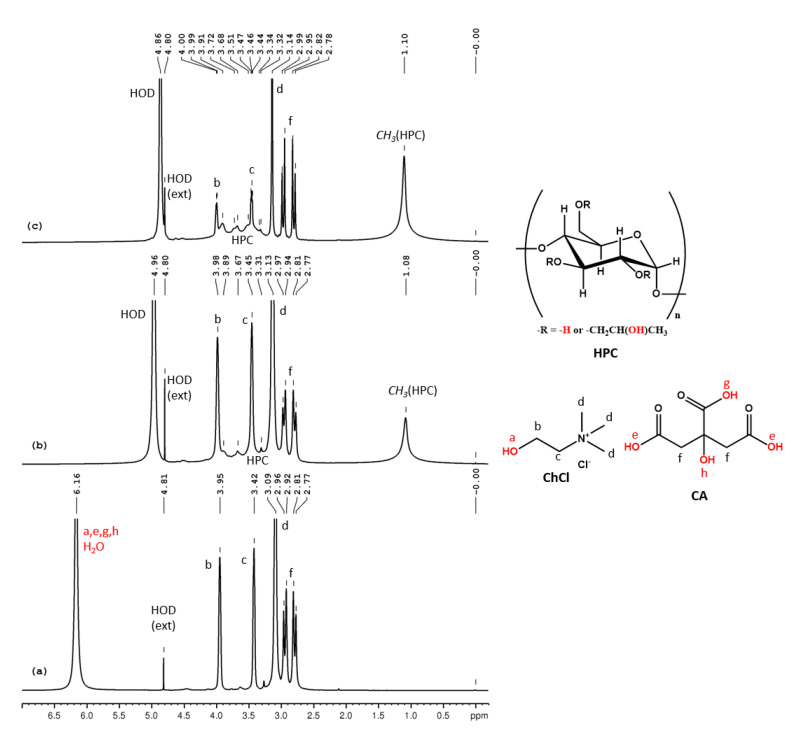
^1^H NMR spectra of ChCl-CA (**a**), HPC-ChCl-CA 29% (**b**) and HPC- ChCl-CA 17% (**c**). In red color are marked the exchangeable protons.

ChCl-CA 100% could not be analyzed by NMR due to its high viscosity. We recorded the ^1^H NMR spectrum (Figure 6a) for a less viscous mixture of 80% ChCl-CA and 20% D_2_O. The assignments of the peaks are (δ, ppm): 6.16 (CA-*COOH*, CA-*OH*, ChCl-*OH*, HOD), 3.95 (CHCl-*CH*_2_-OH), 3.42 (CHCl-*CH*_2_-N), 3.09 (ChCl-N-(*CH*_3_)_3_), 2.94 (CA-*CH*_2_) and 2.79 (CA-*CH*_2_). Since D_2_O was used for the sample preparation, the intrinsic water content of this mixture could not be calculated from NMR data. In the ^1^H NMR spectra for 14% HPC, 29% ChCl-CA and 57% D_2_O (*w*/*w*/*w*) and 16% HPC, 16% ChCl-CA and 66% D_2_O (*w*/*w*/*w*) the peaks for HPC are observed at 1.10 ppm and 3–4.5 ppm (overlapped with ChCl and CA peaks). The peak for HOD and for the labile protons shifts from 6.16 in ChCl-CA to 4.96 and 4.86 ppm in the two samples (Figure 6b,c) due to increasing rate of exchange determined by increasing the amount of water from the system.

### 2.3. Thermal Stability

**Figure 7:**
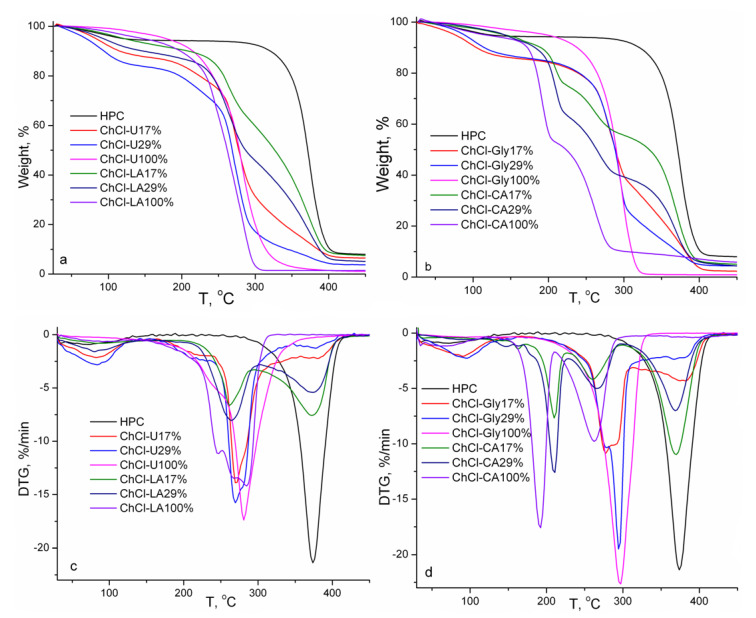
TG and DTG curves of HPC, NADES and HPC-NADES gels (17% and 29% *w*/*w*).

The thermal stabilities and kinetic parameters of pure studied NADES and their gels with HPC are investigated using thermogravimetry and derivative thermogravimetry under dynamic conditions of temperature. The thermogravimetric curves of the studied pure NADES and gel mixtures with HPC (17 and 29% *w*/*w*) are shown in Figure 7. The thermal characteristics obtained from TG and DTG curves are summarized in Table 2.

Generally, the values of initial decomposition temperatures for NADES are between those of pure constituents, HBD and HBA. The thermal stability of HBDs determines the thermal stabilities of the resulting NADES, i.e., the thermal stabilities of NADES increase compared to pure HBDs and become worse compared to HBAs [25,26,27]. The thermal stability of NADES is influenced by hydrogen bonding between HBA and HBD molecules. The degradation stages below 150 °C are associated with evaporation of water. It is evident from Figure 7 and Table 2 that the thermal stability of HPC is superior to those of pure NADES and their mixtures with HPC because the onset degradation temperature of HPC is higher. The thermal degradation is complex because the main degradation peaks are comprised of several processes of thermal degradation.

The non-isothermal kinetic parameters of the thermal degradation for overlapped processes following water evaporation were evaluated in terms of the Coats–Redfern [28], Flynn–Wall [29] van Krevelen [29] and Urbanovici–Segal [30] integral methods (Table 3). The activation energy of thermal degradation is regarded as a semi-quantitative factor of thermal stability. It is evident from Table 3 that the values of activation energy and order of reaction are found higher for NADES and their mixtures with HPC (the degradation stage corresponding to HPC component) based on citric acid and glycerol. This result confirms the importance of the hydrogen bonding network in dictating the thermal stability which is in connection with the FT-IR, DSC results and rheology measurements.

### 2.4. DSC Analysis

**Figure 8:**
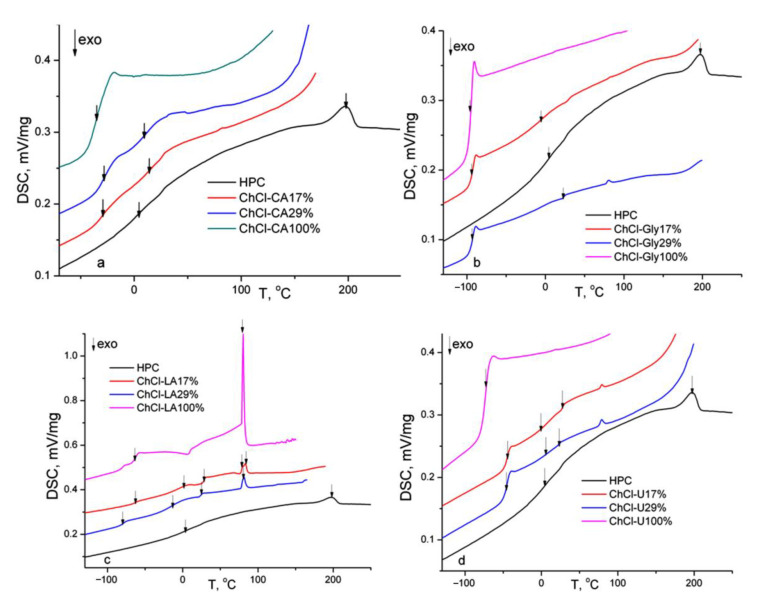
DSC thermograms (second heating runs) of HPC, NADES and HPC-NADES gels (17% and 29% *w*/*w*).

In Figure 8 the DSC thermograms (second heating runs) of the HPC, NADES and HPC-NADES gel mixtures (17% and 29% *w*/*w*) are shown. The thermal characteristics resulting from DSC are summarized in Table 4.

The pure NADES evidence mainly glass transitions, this variant being called low transition temperature mixtures [3]. It is speculated that the low amounts of water determine the increased viscosity and decreased molecular mobility, promoting glass formation instead of crystal [31]. Only ChCl-U reveals melting endotherm at 15 °C (first heating run). Pure ChCl-LA reveals at around 80 °C a solid–solid transition found in case of choline chloride-urea systems for choline chloride rich composition (χ_ChCl_ =0.5–0.67) [31,32]. Multiple glass transitions are observed which is characteristic for phase separated systems. Each phase has its own glass transition. For the ChCl-CA and ChCl-Gly mixtures two glass transitions corresponding to the NADES and HPC component are observed. In the case of ChCl-LA and ChCl-U mixtures three glass transitions are observed. The Tg at highest temperature is found around 28 °C for both mixtures and is attributed to a phase composed mainly of HPC. The Tg of HPC (powder) is found at 21 °C (second heating run, 10 °C/min). The three values of the glass transitions indicate higher degree of phase segregation for ChCl-LA and ChCl-U mixtures due to less strength of the hydrogen bonding. The values of Tgs in mixtures (for the phase rich in NADES) are higher than those of pure NADES suggesting the formation of the hydrogen bonding network. Some polymer complexes have higher Tg values because hydrogen bonds act as physical crosslinks [33]. It was found in literature that the values of T_g_ of mixtures of biomaterials are in connection with the number of hydroxyl groups per molecule [34] intermolecular hydrogen bonding and molecular packing. The decrease of the Tg corresponding to HPC component indicates a plasticization of HPC by NADES.

### 2.5. Rheological Properties

The study of rheological properties of pharmaceutical systems (simple liquids, ointments, creams, pastes, suppositories, suspensions and colloidal dispersing, emulsifying and suspending agents) are very important as a quality control instrument in order to assure product quality and diminished batch-to-batch discrepancies. These studies contribute to the characterization of manufacturing, storage and transport processes, or the behavior during pharmaceutical products’ administration or therapeutic outcome [35,36,37].

#### 2.5.1. Flow Curves

**Figure 9:**
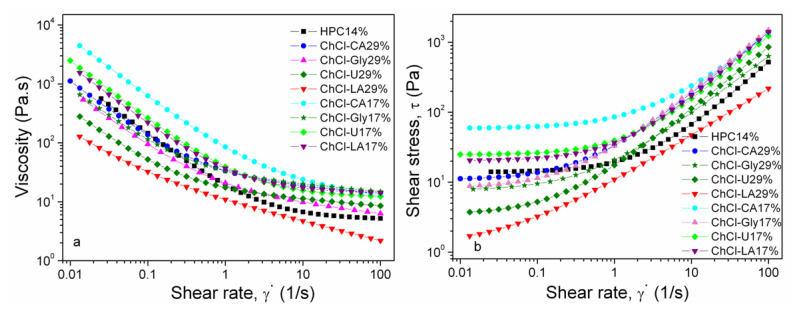
The flow behavior of the HPC (solution 14%) and HPC-based NADES gels (17% and 29%): viscosity (**a**) and shear stress (**b**).

In Figure 9a,b the flow behavior of NADES-HPC gel solutions (17% and 29% *w*/*w* NADES) is illustrated. The rheological plots (log-log scale) of NADES-HPC gel solutions show (Figure 9a) non-Newtonian, shear thinning (pseudoplastic fluid) behavior. The viscosities decrease by adding NADES in solutions in the following order: HPC 14% > ChCl-CA 29% > ChCl-Gly 29% > ChCl-U 29% > ChCl-LA 29%.The rheological behavior of these systems is governed by the formation of an extensive network of inter- and intramolecular interactions such as hydrogen bonding, assuring high cohesive forces in the bulk liquid. The strength of hydrogen bonding and viscosities of the starting NADES influences the resulting viscosity values of the NADES-HPC aqueous solutions. The hydrogen bond density of the NADES is determined by the number of hydrogen bonds donated. The hydrogen bond donor count values for citric acid (4) (https://pubchem.ncbi.nlm.nih.gov/compound/Citric-acid), glycerol (3) (https://pubchem.ncbi.nlm.nih.gov/compound/Glycerol), urea (2) (https://pubchem.ncbi.nlm.nih.gov/compound/Urea) and lactic acid (2) https://pubchem.ncbi.nlm.nih.gov/compound/Lactic-acid) are in agreement with viscosity, thermal stability, DSC and FT-IR results.

The addition of water or polymers changes the flow behavior and internal resistance of the resulting systems. This result can be corroborated with IR, DSC and TGA results. In case of NADES or ionic liquids shear thinning is associated with breaking of the hydrogen bonds. Electrostatic interactions and the re-arrangement of the ions could contribute to shear thinning as well. In case of polymers the disentanglement of the polymer coils and alignment of the polymer chain into the flow direction are also associated with shear thinning phenomenon.

From Figure 9b it is evident that the studied solutions exhibit yield stress (*τ_0_*) which must be exceeded prior to deformation or flowing of the fluid [38]. The yield stress is obtained through extrapolation of the flow curve at low shear rates to zero shear rate. For shear stress values below the yield stress values the fluid behaves as a rigid solid. Usually, the yield stress is considered as the transition stress between elastic solid-like behavior and viscous liquid-like behavior and is connected to the characteristic network structure. When external stress is greater than yield stress the flow curve does not pass through origin and may be linear or non-linear. For a non-Newtonian fluid the shear stress vs. shear rate curve is non-linear or does not pass through the origin. Shulman model [39] was chosen to characterize the shear stress vs. shear rate curves:

$$\tau ={\left[\tau_{0}^{1/n}+{\left(\mu \gamma \cdot \right)}^{1/m}\right]}^{n}$$

where *τ* is shear stress, *τ_0_* is the yield stress, *μ* is the plastic viscosity (Pa..s), *K* is consistency index (Pa.s^n^), *γ^.^* is shear rate and *m*, *n* are power exponents related to material properties. The following simpler rheological models derived by reducing the coefficients were applied for the studied shear stress-shear rate curves:

1. Bingham model [40]:

(1) $$\tau =\tau_{0}+\mu \gamma$$

2. Herschel–Bulkley model [41]:

(2) $$\tau =\tau_{0}+K\gamma \cdot^{n}$$

3. Vocadlo model [42]

(3) $$\tau ={\left(\tau_{0}^{1/n}+K\gamma \cdot \right)}^{n}$$

4. Casson model [43]

(4) $$\tau^{0.5}=\tau_{0}^{0.5}+{\left(\mu \gamma \cdot \right)}^{0.5}$$

When the flow index (dimensionless) *n* =1 the rheological models reduce to the Bingham model. For *n* < 1 the system is non-Newtonian pseudoplastic (shear thinning) whereas for *n* > 1 (unusual) [44] the system is shear thickening (dilatant). A lower value of *n* indicates a more non-Newtonian shear thinning fluid (increased pseudoplasticity). The behavior of NADES is mainly Newtonian and non-Newtonian when they are similar to ionic liquids [45]. The rheology of HPC gels is well described by Herschel-Bulkley model [46]. The values of *n* close to 1 (Herschel Bulkley model in the Table 5) indicate that Bingham model is more appropriate. The values of *K* serve as the viscosity indices of the systems. 

The value of *τ*_0_ refers to the amount of minimum stress necessary for disrupting the networked structure in order to initiate the flow. The yield stress gives information on chain rigidity, hydrogen bonding and molecular weight being connected to viscosity values [47]. The calculated values of *τ_0_* are in good agreement with the experimental ones. The values of *τ_0_* are in the same order as viscosity for the studied samples (Figure 9b). 

By increasing the value of exponent >0.5 in the Casson equation approaching the Bingham model the fitting results are closer to those experimentally found.

#### 2.5.2. Dynamic Oscillatory Measurements

Viscoelastic properties of HPC-NADES solutions were studied by oscillation. The elastic modulus is linked with the energy stored in elastic deformation whereas the viscous modulus is linked with viscous dissipation effects.

**Figure 10:**
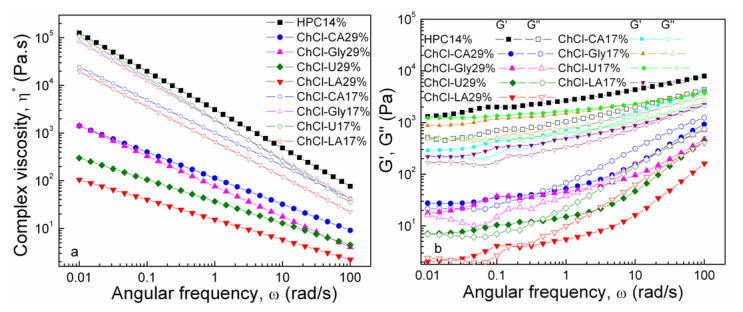
Complex viscosity and dynamic moduli, *G*′ and *G*′′ as functions of angular frequency at 20 °C.

In Figure 10 the dependences of complex viscosity and dynamic moduli, G’ and G” on angular frequency for the studied solutions are illustrated.

Oscillatory measurements are preferred instead of steady shear measurements in order to avoid the disruption of the networks [48]. The departure from the Cox–Merz rule [49]

(5) $$\eta \left(\gamma \cdot \right)≅{\left|\eta *\left(\omega \right)\right|}_{\omega }=\gamma$$

can be explained by the structural damage caused by the excessive shear during flow curves measurements. The complex viscosity decreases at increased concentration of NADES.

The property of viscoelasticity is important in polymeric solutions because they show both liquid and solid-like properties. The extent of intermolecular association/aggregation and chain entanglement determine the relative contributions of the elastic and viscous elements.

The viscoelastic response of the samples is influenced both by composition and concentration of NADES with visible differences in behavior between the two series of samples. It can be noticed that HPC 14% has an elastic (solid-like) behavior (*G*′ > *G*′′) over the entire frequency domain studied. The capability of the polymer network to store the imposed energy increases and the behavior is more elastic (solid-like). A similar pattern of behavior is observed for all the mixtures of 17% concentration, with high, but quite close values measured for *G*′ and *G*′′.

In the case of 29% concentration series, the storage (*G*′) and loss (*G*′′) moduli reveal a modified behavior and much lower values as compared with HPC and the 17-coded samples series. All 29%-compositions show cross-over points when the elastic component outweighs the viscous ones, which indicate transition from a predominantly liquid-like behavior (*G*′′ > *G*′) at increased frequencies towards an elastic/solid-like response at low ω. The cross-over point is located at characteristic frequency the reciprocal of which represents a measure of the relaxation time of the polymer network. In case of polymeric systems the relaxation time *λ* under dynamic shear can be determined by means of the following equation [50,51]:

(6) $$\lambda =\frac{G\prime }{\eta^{*}\omega^{2}}$$

where *G*′ is the storage modulus, *ω* is the angular frequency and η* is complex viscosity. The systems with high values of complex viscosity correspond to early relaxations of polymer chains (short relaxation times).

Increasing trend of ω and *G*′ = *G*′′ values at crossover point:

ChCl-LA 29% − ω = 0.1668//0.02132 rad/s and *G*′ = *G*′′ = 4.036//2.186 Pa

ChCl-U 29% − ω = 0.3683 rad/s and *G*′ = *G*′′ = 12.20 Pa

ChCl-CA 29% − ω = 0.3706 rad/s and *G*′ = *G*′′ = 41.24 Pa

ChCl-Gly 29% − ω = 2.145 rad/s and *G*′ = *G*′′ = 54.88 Pa

#### 2.5.3. Effect of Temperature

**Figure 11:**
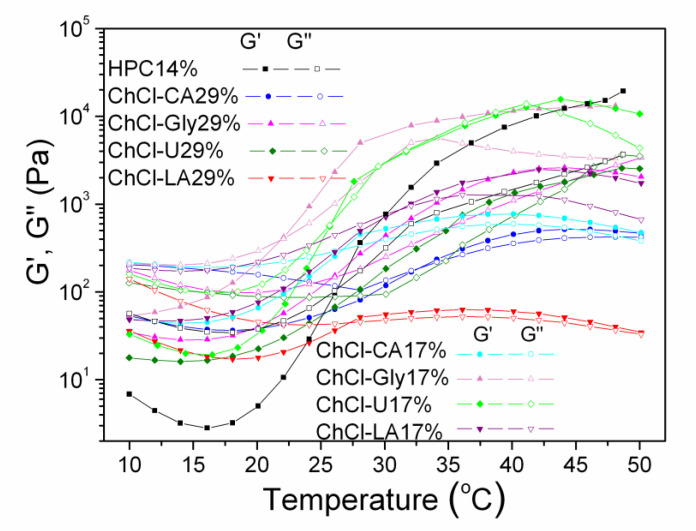
Dynamic moduli, *G*′ and *G*′′ versus temperature for studied HPC-NADES solutions.

The influence of temperature was investigated by time-temperature sweep measurements performed from 10 to 50 °C with a heating rate of 2 °C/min (Figure 11). The storage modulus (*G*′), loss modulus (*G*′′) and complex viscosity (η*) were recorded at frequency of 1 Hz, applying a constant stress (*τ*) value corresponding for each sample in the regime of linear viscoelasticity.

All samples show thermothickening properties. At low temperatures, generally below 20 °C, the mixtures show fluid/viscous-like behavior with a weak elastic contribution. Above 20 °C a sol-gel transition takes place, with increase of elasticity, mainly between 20–50 °C. The transition is sharper for HPC 14%, HPC-ChCl-Gly 29% and HPC-ChCl-U 29% and smoother for HPC-ChCl-CA29% and HPC-ChCl-LA29% depending on the composition of the sample. Solutions behave as elastic gels above 40 °C, but a new crossover point occurred around a dissolution temperature (T_dis_) for HPC-ChCl-U 29% and HPC-ChCl-Gly 29%.

The following cross-over points are observed for the studied solutions:

HPC-T_gel_ = 28.6 °C; *G*′ = *G*′′ = 642.6 Pa

ChCl-CA 17% T_gel_ = 26.43 °C; *G’* = *G*′′ = 304.87 Pa

ChCl-Gly 17% T_gel_ = 21.87 °C; *G*′ = *G*′′ = 384.00 Pa

ChCl-U 17% T_gel_ = 25.47 °C; *G*′ = *G*′′ = 575.71 Pa

ChCl-LA 17% T_gel_ = 31.19 °C; *G*′ = *G*′′ = 888.78 Pa

ChCl-CA 29% T_gel_ = 32.1 °C; *G*′ = *G*′′ = 186.9 Pa

ChCl-LA 29% T_gel_ = 27.3 °C; *G*′ = *G*′′ = 43.1 Pa

ChCl-Gly 29% T_gel_ = 26.2 °C: *G*′ = *G*′′ = 142.8 Pa; T_dis_= 47.2 °C; *G*′ = *G*′′ = 2611.3 Pa

ChCl-U 29% T_gel_ = 28.3 °C; *G*′ = *G*′′ = 94.8 Pa; T_dis_ =45.4 °C; *G*′ = *G*′′ = 2059.8 Pa

It is well known that HPC exhibits a lower critical solution *temperature* (LCST) at 41–43 °C in aqueous media [52,53].

The phase separation from solution and solidification above a certain temperature, LCST or “cloud point” when water becomes a poor solvent and the solution becomes cloudy is characteristic for thermoresponsive hydrogels (negative temperature-sensitive hydrogels). Below LCST the polymers are soluble and above LCST they become more and more hydrophobic and insoluble with gel formation. Hydrogen bonding and hydrophobic interactions are dependent on temperature and create phase separation. The increase of moduli with increasing temperature is correlated with the structure formation as phase separation and gelation occur nearly at the same time [54]. The LCST behavior is modified for NADES-HPC solutions. Moreover, T_dis_ is associated with upper critical solution temperature behavior (UCST-positive temperature-sensitive hydrogels). Above UCST the polymers are soluble.

### 2.6. Antibacterial and Antifungal Activities

It is crucial to characterize the biological properties of NADES prior to their industrial applications especially in drug delivery systems, pharmaceutical and food-related applications. Recently there have been reported studies on the development of deep eutectic solvents with antibacterial properties [55,56,57]. Depending on the NADES’s composition different antibacterial and antifungal activities are found. The components of NADES give the antibacterial properties to the complex and can manifest synergistic effects [55,56,57,58,59,60,61]. The increased antibacterial effect of NADES with acids was reported [55,62]. Table 6 evidences the antibacterial and antifungal activities of the tested NADES towards common pathogen bacteria and yeast strain which were determined by means of disc diffusion method [63,64].

The diameters of the inhibition zones (in mm) corresponding to the tested compounds are shown in Table 6. Results are expressed as means ± SD. It is obvious from Table 6 that the order of antibacterial and antifungal activities is as follows: ChCl-CA > ChCl-LA > ChCl-U > ChCl-Gly, evidencing the higher antibacterial and antifungal activities given by acids. The antibacterial and antifungal activities in the mixtures with HPC give lower values of the diameters of the inhibition zones.

**Figure 12:**
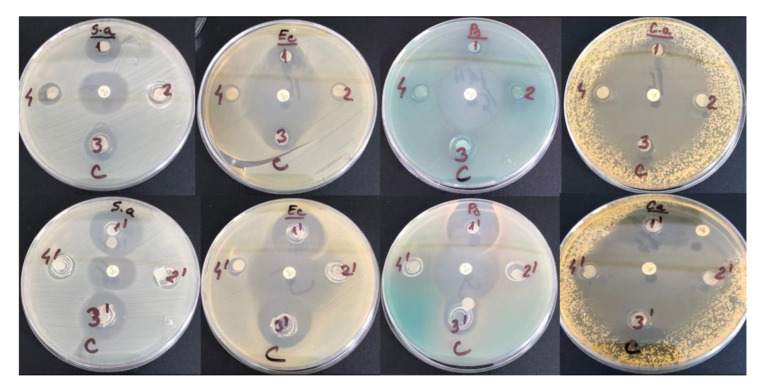
Antimicrobial activity of tested compounds HPC-ChCl-CA 29% (1) and ChCl-CA 100% (1′); HPC-ChCl-Gly 29% (2) and ChCl-Gly 100% (2′); HPC-ChCl-LA 29% (3) and ChCl-LA 100% (3′); HPC-ChCl-U 29% (4) and ChCl-U 100% (4′) against *Staphylococcus aureus* ATCC 25923, *E. coli* ATCC 25922, *Pseudomonas aeruginosa* ATCC 27853 and *Candida albicans* ATCC 90028.

The inhibition zones of the studied samples against *S. aureus*, *E. coli*, *Pseudomonas aeruginosa* and *C. albicans* are shown in Figure 12.

### 2.7. Evaluation of Biocompatibility

**Figure 13:**
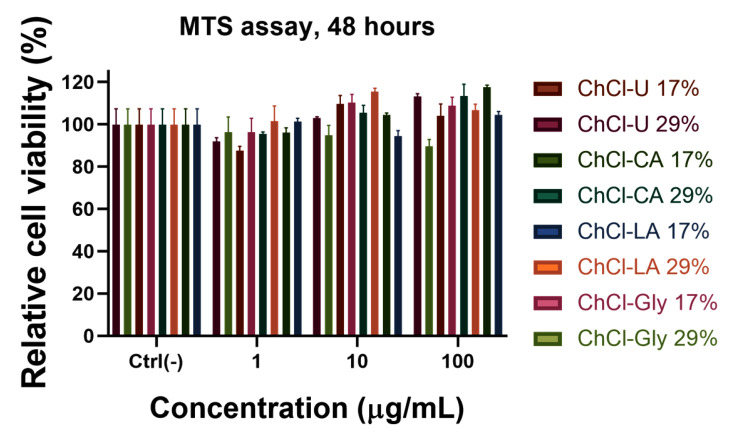
MTS assay on human gingival fibroblast of the prepared HPC-NADES gels.

Biocompatibility of NADES gels mixture depends on the structure of the mixture components. In general, choline chloride showed a lower cytotoxicity than many other ionic liquids, such as imidazolium or pyridinium [65]. Based on the principle of green chemistry, two characteristics, biocompatibility and biodegradability, are necessary to be investigated before that they can be named “green solvents” or co-solvents for pharmaceutical applications. NADES possess a high potential to improve drug development and release. For use as pharmaceutical excipients these systems must be investigated for their toxicological effects by evaluation of cytotoxicity in human cell line [66]. In order to evaluate the biocompatibility with normal cells (human gingival fibroblast) of the obtained HPC-NADES gels, MTS assay was performed, which allows the estimation of the cell viability and proliferation (Figure 13). The compounds were tested at three concentrations: 1 μg/mL, 10 μg/mL and 100 μg/mL. The results indicated a very good compatibility at all studied concentrations, the cell viability being almost 100%, excepting the NADES gels based on ChCl-CA with a cell viability of 90%. The MTS test is an indirect colorimetric method for estimating cell viability and proliferation. The reagent contains a tetrazolium compound that is transformed by the mitochondria of healthy cells into a colored formazan. The more living cells there are, or the more intense the mitochondrial metabolism, the more formazan will be obtained. Thus, in our case, a stronger signal than in the untreated sample may mean a more intense proliferation or a stimulation of the mitochondrial metabolism. Choline chloride component is mainly responsible for the higher viability, this being a part of the vitamin B complex.

## 3. Conclusions

In this work NADES-HPC gels were prepared. FT-IR, ^1^HNMR, DSC, TGA and rheology data revealed that hydrogen bond acceptor–hydrogen bond donor interactions of the studied NADES, their concentration and water content considerably influence the physico-chemical characteristics of the studied systems. The peak at 955 cm^−1^ in the IR spectra attributed to ammonium structure in NADES vanishes for lower content of NADES in solutions for the systems with increased strength of hydrogen bonding. HPC-NADES gel compositions have thermal stabilities lower than HPC and higher than NADES components. The thermal stability was investigated by means of activation energy and order of reaction. Thermal transitions reveal multiple glass transitions characteristic for phase separated systems. Increased strength of hydrogen bonding network was obtained for citric acid and glycerol-based systems compared to urea and lactic acid-based systems. Flow curves evidence shear thinning behavior. Bingham, Herschel–Bulkley, Vocadlo and Casson rheological models were employed to fit the rheological data. The studied solutions evidence thermothickening behavior because of specific LCST behavior of HPC in aqueous solutions. All prepared NADES-HPC gels proved to have a very good biocompatibility with HGF normal cell line. *S. aureus*, *E. coli*, *P. aeruginosa* and *C. albicans* were tested to assess the antibacterial and antifungal activities of the investigated systems. The order of antibacterial and antifungal activities is as follows: citric acid >lactic acid > urea > glycerol content in NADES-HPC gels.

## 4. Experimental Part

### 4.1. Materials

Hydroxypropyl cellulose (Klucel LF M_w_ 95,000 Da) was purchased from Aqualon, Hercules Inc., Wilmington, NC, USA and used as received. Choline chloride, ≥98%, was purchased from (Sigma-Aldrich, Merck Romania SRL, Bucharest, Romania, an affiliate of Merck KGaA, Darmstadt, Germany ), urea was purchased from (Sigma-Aldrich, Merck Romania SRL, Bucharest, Romania, an affiliate of Merck KGaA, Darmstadt, Germany), citric acid (99%)was purchased from (Sigma-Aldrich, Merck Romania SRL, Bucharest, Romania, an affiliate of Merck KGaA, Darmstadt, Germany), DL-lactic acid (90%)was purchased from (Sigma-Aldrich, Merck Romania SRL, Bucharest, Romania, an affiliate of Merck KGaA, Darmstadt, Germany) and glycerol was purchased from Merck Romania SRL, Bucharest, Romania, an affiliate of Merck KGaA, Darmstadt, Germany). Choline chloride and urea were dried under vacuum for several days before use.

### 4.2. Preparation of Aqueous HPC-NADES Gel Solutions

HPC solutions (14% *w*/*v*) were prepared by solving HPC in distilled water. The solutions were kept on a magnetic stirrer at room temperature and were stirred at moderate speed for several hours. Original NADES were prepared by mixing the two initial compounds (choline chloride with the corresponding HBD component: urea, glycerol, lactic acid or citric acid) with added small amounts of water at 60–80°C until homogeneous liquid was obtained, then the mixtures were dried in oven at 25 °C. HPC-NADES aqueous solutions 17% and 29% were obtained by gradual addition of NADES at RT to HPC solution 14%. The compositions (wt%) of the studied HPC-NADES aqueous solutions are shown in Table 7.

### 4.3. Measurements

ATR (attenuated total reflection infrared)-IR (infrared) spectra were measured with an equipment Bruker Vertex 70 having an ATR accessory module equipped with a ZnSe crystal. The IR spectra were performed at room temperature with accumulation of 32 scans. The resolution of the registered spectra was 4 cm^−1^.The O-H and N-H spectral regions in the 3800–3000 cm^−1^ spectral range were deconvoluted with the curve-fitting function accessible from OPUS 6.5 software (Bruker, Ettlingen, Germany). The maxima peak’s position was determined by second derivative of the spectra and used to calculate the energy of the hydrogen bonding and the H-bond distance according to a procedure already described [19].

NMR spectra were recorded on a Bruker Avance NEO 400 MHz Spectrometer equipped with a 5-mm QNP direct detection probe and z-gradients, using standard parameter sets provided by Bruker. The ^1^H NMR and ROESY spectra were recorded at room temperature in presence of external D_2_O containing TSP (sodium salt of trimethylsilyl propionic acid) and were calibrated on the TSP peak (0 ppm). NMR sample preparation: 0.6 mL of each pure NADES mixture were transferred into NMR tubes and prior to recording the spectra, in each tube a capillary containing D_2_O and TSP was added. For HPC-NADES 17% mixtures: a HPC solution in D_2_O (14% *w*/*w*) was prepared by dissolving 0.68 g HPC in 3 mL D_2_O; in 0.85 g of this HPC in D_2_O solution, 0.68 g of NADES were slowly added, stirred for homogenization and transferred into NMR tubes. Prior to recording the spectra, in each tube a capillary containing D_2_O and TSP was added. For each of the HPC-NADES 29% mixtures: 0.43 g mixture was weighted and then dissolved in 0.52 mL D_2_O. The resulting solutions were transferred into NMR tubes and prior to recording the spectra capillaries containing D_2_O and TSP were added.

STA 449F1 Jupiter NETZSCH (NETZSCH Analysing and Testing, Netzsch, Germany) equipment for investigation of thermal stability of the HPC/NADES blends was used. The measurements were conducted in the 30–700 °C temperature range, in 50 mL min^−1^ nitrogen flow. The heating rate was 10 °C min^−1^. 

DSC 200 F3 Maia equipment (Netzsch, Germany) operating under nitrogen 50 mL min^−1^ flow in heating/cooling rates of 10 °C min^−1^ was used to analyze the NADES/HPC blends. About 10 mg of each sample were heated in aluminum crucibles with pierced and pressed lids from −150 °C to 250 °C. Before TGA and DSC analyses, the HPC-NADES aqueous solutions were dehydrated at room temperature for several days followed by drying under vacuum for several days.

The rheological behavior study was accomplished by using an Anton PaarPhysica MCR 301 Rheometer (Anton Paar, Austria), equipped with a 50 mm diameter cone-plate geometry with a 1° angle. Steady shear flow and dynamic oscillatory measurements on HPC-NADES aqueous solutions were carried out at 25 ± 0.1 °C. A Peltier heating system was used for precise temperature control. Flow measurements were performed over the shear rate range 0.05–200 1/s. For the oscillatory shear tests, the logarithmic frequency sweeps were carried out over the angular frequency range 200–0.1/0.05 rad/s. Preliminary strain sweep tests done at 10 rad/s over the strain range 0.01–200%confirmed that the tests were in the linear viscoelastic regime (LVE). Temperature sweep measurements were carried out at a constant shear rate of 20 1/s, over the temperature range between 10 to 50 °C at a heating rate of 2 °C/min. Prior each measurement, after loading, the sample was held for certain time, as previously tested, to permit stress relaxation and temperature equilibration.

#### 4.3.1. Antimicrobial Susceptibility Tests

The antimicrobial activity was studied using Gram positive bacteria (*Staphylococcus aureus* ATCC 25923), Gram negative bacteria (*Escherichia coli* ATCC 25922, *Pseudomonas aeruginosa* ATCC 27853) and a pathogenic yeast (*Candida albicans* ATCC 90028).

The antimicrobial activity was assessed by employing the disk diffusion methods [63,64].

*Disc-diffusion method*. Mueller–Hinton agar (Oxoid) and Mueller–Hinton agar Fungi (Biolab) were inoculated with the suspensions of the tested microorganisms: *Staphylococcus aureus* ATCC 25923, *Escherichia coli* ATCC 25922, *Pseudomonas aeruginosa* ATCC 27853 and *Candida albicans* ATCC 90028. Sterile stainless-steel cylinders (5 mm internal diameter; 10 mm height) were applied on the agar surface in Petri plates. Then, 100 µL of the tested compounds were added into cylinders. The plates were left 10 min at room temperature to ensure the equal diffusion of the compound in the medium and then incubated at 35 °C for 24 h. As reference antimicrobial drugs commercially available discs were used containing Ciprofloxacin (5 µg/disk), Fluconazole (25 μg/disk) and Voriconazole (1 μg/disk). After incubation, the diameters of inhibition were measured. All assays were carried out in triplicate.

#### 4.3.2. MTS Assay

Human gingival fibroblast (HGF) cells were cultured in a complete medium containing alpha-MEM, 10% FBS and 1% penicillin-streptomycin-amphotericin B mixture. For cell culture the cells were maintained in a humidified environment with 5% CO_2_ at 37 °C. After the cells were multiplied sufficiently, the culture medium was removed and the cells were washed with phosphate buffer and then detached with Tryple.

For MTS assay, 25 × 10^5^ cells/well were seeded into 24-well plates and incubated over night. The next day, the HPC-NADES mixtures (30 mg) were diluted at three concentrations and then placed on the top of each well previously seeded with cells. The thus prepared plates were returned to the incubator.

After 48 h, plates were treated with MTS reagent according to manufacturer’s protocol and the obtained formazan was quantified after 2 h by measuring the absorbance at 490 nm with a plate reader (BMG LABTECH, Ortenberg, Germany). The cell viability was estimated as the absorbance of the samples as a percentage of the absorbance of the untreated cells.

## Figures and Tables

**Table 1:** The energy and the distance of hydrogen bonds in NADES and HPC-NADES samples.

Sample	HPC-NADES (Dry State)		HPC-NADES (in Solution)	
	E_H_ (kJ)	R(Å)	E_H_ (kJ)	R(Å)
HPC	27.832	2.753	15.318	2.792
HPC-ChCl-U 17%	20.401 (N-H)	2.717 (N-H)	14.885 (N-H)	2.851 (N-H)
	22.151 (O-H)	2.769 (O-H)	20.281 (O-H)	2.776 (O-H)
HPC-ChCl-U 29%	20.552 (N-H)	2.714 (N-H)	15.860 (N-H)	2.827 (N-H)
	22.366 (O-H)	2.770 (O-H)	20.568 (O-H)	2.775 (O-H)
HPC-ChCl-Gly 17%	21.863	2.771	20.065	2.777
HPC-ChCl-Gly 29%	20.281	2.776	20.136	2.776
HPC-ChCl-LA17%	19.633	2.778	19.921	2.777
HPC-ChCl-LA 29%	18.482	2.782	19.489	2.778
HPC-ChCl-CA 17%	18.770	2.781	18.627	2.782
HPC-ChCl-CA 29%	16.397	2.788	18.770	2.781
Original NADES	in dry state		in solution	
ChCl-U	21.006 (N-H)	2.703 (N-H)	16.623 (N-H)	2.808 (N-H)
	23.373 (O-H)	2.766 (O-H)	21.001 (O-H)	2.774 (O-H)
ChCl-Gly	24.092	2.764	22.150	2.770
ChCl-LA	22.798	2.768	20.424	2.775
ChCl-CA	21.575	2.772	18.771	2.781

**Table 2:** The thermal characteristics from TG and DTG curves of HPC, NADES and HPC-NADES gels (17% and 29% *w*/*w*).

Sample	Stages, °C	T_max_^a^, °C	Δw ^b^, %
ChCl-CA 100%	I 36.8–121.6	61.7	6.8
	II 171.7–200.9	191.6	40.9
	III 225.5–277.2	262.5	41.2
	IV 307.8–419.0	364.3	3.2
ChCl-Gly 100%	I 109.7–176.9	128.0	5.5
	II 228.2–318.9	296.3	92.5
ChCl-LA 100%	I 74.5–143	89.1	4.87
	II 171.3–232.9	206.1	14.5
	III 232.9–315.0	284.6	78.1
ChCl-U 100%	I 127.9–223.2	179.0	10.9
	II 223.2–269.3	237.0	24.8
	III 269.3–320.8	280.7	62.4
HPC	I 26.7–122.7	72.6	6.7
	II 306.3–399.7	374.0	85.1
ChCl-CA 17%	I 157.3–177.2	162.8	8.9
	II 194.4–216.2	210.4	16.6
	III 236.6–282.7	260.5	18.2
	IV 328.2–391.6	369.5	51.8
ChCl-Gly 17%	I 43.5–120.4	90.8	17.2
	II 249.3–301.5	277.3	47.8
	III 334.7–398.6	382.2	31.9
ChCl-LA 17%	I 61.6–143.3	89.8	9.48
	II 231.4–280.0	261.6	27.3
	III 301.3–396.3	372.2	55.8
ChCl-U 17%	I 36.3–121.6	88.0	12.8
	II 184.3–259.1	214.0	14.4
	III 259.1–298.2	270.5	39.6
	IV 298.2–404.3	380.1	25.7
ChCl-CA 29%	I 69.7–135.5	90.0	3.4
	II 135.5–156.8	147.2	4.95
	III 191.7–216.8	210.1	28.8
	IV 238.5–285.3	266.1	23.0
	V 320.7–391.6	368.9	35.3
ChCl-Gly 29%	I 55.5–126.9	95.8	15.9
	II 249.7–287.0	278.2	31.1
	III 287.0–303.5	295.0	26.7
	IV 303.5–393.7	366.7	20.6
ChCl-LA 29%	I 40.9–125.8	79.8	12.1
	II 223.3–281.1	264.4	40.2
	III 298.5–398.6	374.2	42.3
ChCl-U 29%	I 38.9–116.6	83.7	15.5
	II 182.1–247.5	206.9	13.5
	III 254.6–292.6	269.4	57.3
	IV 334.9–402.9	377.6	8.4

^a^ Temperature corresponding to maximum rate of decomposition. ^b^ Weight loss percentage corresponding to degradation stage.

**Table 3:** Kinetic parameters of thermal degradation of HPC, NADES and HPC-NADES gels (17 and 29% *w*/*w*).

Sample	Activation Energy (kJ/mol)/ln A/Order of Reaction			
	Coats–Redfern	Flynn–Wall	Van Krevelen	Urbanovici–Segal
ChCl-CA 100%	II 367.1/92.13/1.7	356.2/90.35/1.7	382.2/96.0/1.7	354.9/88.8/1.5
	III 211.2/43.69/1.3	209.0/43.26/1.3	233.0/48.72/1.4	214.2/44.43/1.3
ChCl-Gly 100%	163.98/30.17/1.1	164.5/30.32/1.1	182.8/34.2/1.1	165.67/30.6/1.1
ChCl-LA 100%	96.8/16.31/0.9	100.2/17.38/0.9	120.8/21.74/1.0	96.1/16.18/0.8
ChCl-U 100%	51.3/4.86/0.1	55.8/6.84/0.0	70.4/9.42/0.4	53.4/5.55/0.2
HPC	222.2/36.71/1.1	221.1/36.53/1.1	251.3/42.23/1.2	224.0/37.1/1.1
ChCl-CA 17% ^a^	305.7/53.15/1.4	300.6/52.37/1.4	337.8/59.25/1.5	309.3/53.89/1.4
ChCl-Gly 17% ^a^	235.8/39.55/1.4	234.3/39.3/1.4	263.7/44.84/1.5	239.9/40.37/1.4
ChCl-LA 17% ^a^	160.4/25.64/1.3	156.8/24.96/1.2	178.6/29.09/1.3	157.2/25.0/1.2
ChCl-U 17% ^a^	186.6/30.47/1.4	187.4/30.67/1.4	213.9/35.7/1.5	189.8/31.16/1.4
ChCl-CA 29% ^a^	192.4/31.60/1.2	192.8/31.70/1.2	214.0/35.65/1.2	194.4/32.03/1.2
ChCl-Gly 29% ^a^	108.1/15.57/1.1	112.6/16.78/1.1	122.6/18.36/1.1	111.2/16.26/1.1
ChCl-LA 29% ^a^	150.8/23.79/1.3	153.2/24.36/1.3	168.9/27.22/1.3	153.3/24.35/1.3
ChCl-U 29% ^a^	234.0/39.27/1.5	232.6/39.04/1.5	252.7/42.71/1.5	237.3/39.95/1.5

^a^ The kinetic parameters were evaluated for thermal degradation stage corresponding to HPC component.

**Table 4:** DSC results (second heating runs) of HPC, NADES and HPC-NADES gels (17% *w*/*w*).

Sample	T_g_, °C	T_m_, °C	Sample	T_g_, °C	T_m_, °C
ChCl-CA 100%	−34.1	-	ChCl-LA 100%	−64.7	-^a^
HPC-ChCl-CA 17%	−28.7; 13.9	-	HPC-ChCl-LA 17%	−63.5;0.8; 28.2	-^b^
HPC-ChCl-CA 29%	−27.6; 9.9	-	HPC-ChCl-LA 29%	−79.5; −14.5; 24.2	-
ChCl-Gly 100%	−96.2	-	ChCl-U 100%	−73.7	-
HPC-ChCl-Gly 17%	−93.3; −5.5	-	HPC-ChCl-U 17%	−44.8; −0.2; 28.1	-
HPC-ChCl-Gly 29%	−93.9; 23.7	-	HPC-ChCl-U 29%	−45.7; 5.0; 23.5	-
HPC	4.8	197.2	-	-	-

^a^ 80.0 °C attributed to solid-solid transition; ^b^ 79.5/83.1 °C attributed to solid-solid transition.

**Table 5:** The shear stress-shear rate curves coefficients of HPC (solution 14%) and HPC-NADES 17% and 29%.

Sample	Bingham	Herschel-Bulkley	Vocadlo	Casson	τ_0exp_, Pa
HPC 14%	τ_0_ = 14.78; μ = 5.11; R = 0.999	τ_0_ = 13.90; μ = 4.93; n = 0.97; R = 0.9997	τ_0_ = 13.89; μ = 6.40; n = 0.96; R = 0.9999	τ_0_ = 14.00; μ = 3.70; R = 0.9957	14.18
ChCl-Gly 29%	τ_0_ = 18.18; μ = 6.44; R = 0.99648	τ_0_ = 15.00; μ = 6.49; n = 1.04; R = 0.98892	τ_0_ = 7.81; μ = 34.06; n = 0.79; R = 0.99989	τ_0_ = 5.77; μ = 5.38; R = 0.99964	10.62
ChCl-CA 29%	τ_0_ = 23.92;μ = 14.22; R = 0.99859	τ_0_ =24.40; μ = 11.35; n = 1.09; R = 0.9902	τ_0_ =11.74; μ = 30.10; n = 0.91; R = 0.99971	τ_0_ = 6.67; μ = 12.29; R = 0.99974	13.70
ChCl-U 29%	τ_0_ = 12.07; μ = 8.72; R = 0.99848	τ_0_ = 3.40; μ = 14.30; n = 0.88; R = 0.99998	τ_0_ = 3.63; μ = 21.76; n = 0.88; R = 0.99998	τ_0_ = 2.21; μ = 7.96; R = 0.99976	5.38
ChCl-LA 29%	τ_0_ = 10.55; μ = 2.31; R = 0.98363	τ_0_ = 1.10; μ = 9.96; n = 0.66; R = 0.99994	τ_0_ = 1.53; μ = 38.74; n = 0.65; R = 0.9999	τ_0_ = 2.88; μ = 2.02; R = 0.9924	3.33
ChCl-CA 17%	τ_0_ = 79.95;μ = 12.57; R = 0.9961	τ_0_ = 62.70; μ = 17.92; n = 0.97; R = 0.9912	τ_0_ = 59.01; μ = 208.06; n = 0.71; R = 0.9996	τ_0_ = 80.00; μ = 8.17; R = 0.9989	64.53
ChCl-Gly 17%	τ_0_ = 23.81;μ = 15.22; R = 0.9984	τ_0_ = 11.50; μ = 17.18; n = 1.02; R = 0.9926	τ_0_ = 8.87; μ = 39.49; n = 0.88; R = 0.9999	τ_0_ = 23.00; μ = 13.59; R = 0.9998	11.68
ChCl-U 17%	τ_0_ = 28.67;μ = 12.17; R = 0.9998	τ_0_ = 27.50; μ = 9.65; n = 1.09; R = 0.9936	τ_0_ = 24.82; μ = 19.27; n = 0.94; R = 0.9999	τ_0_ = 28.00; μ = 9.15; R = 0.9969	26.24
ChCl-LA 17%	τ_0_ = 24.38;μ = 13.99; R = 0.9998	τ_0_ = 22.60; μ = 11.43; n = 1.09; R = 0.9944	τ_0_ = 20.45; μ = 19.78; n = 0.95; R = 0.9999	τ_0_ = 24.00; μ = 11.16; R = 0.9978	22.56

**Table 6:** Antibacterial and Antifungal Activities of the Tested Compounds.

Compounds	Diameter of Inhibition Zones (mm)			
	*S. aureus* ATCC 25923	*E. coli* ATCC 25922	*Pseudomonas aeruginosa* ATCC 27853	*C. albicans* ATCC 90028
ChCl-CA 29%	20.30 ± 0.57	19.00 ± 0.00	0.00	17.50 ± 0.50
ChCl-Gly 29%	0.00	0.00	0.00	0.00
ChCl-LA 29%	16.00 ± 0.00	15.10 ± 0.05	0.00	18.30 ± 0.57
ChCl-U 29%	10.10 ± 0.05	0.00	0.00	12.00 ± 0.00
ChCl-CA 100%	29.00 ± 0.00	26.00 ± 0.00	30.00 ± 0.00	28.00 ± 0.00
ChCl-Gly 100%	0.00	0.00	0.00	13.00 ± 0.00
ChCl-LA100%	20.00 ± 0.00	23.00 ± 0.00	15.00 ± 0.00	21.10 ± 0.05
ChCl-U 100%	12.00 ± 0.00	0.00	0.00	15.00 ± 0.00
Ciprofloxacin (5 µg/disc)	26.70 ± 0.06	30.00 ± 0.00	32.00 ± 0.00	* NT
Fluconazol (25 µg/disc)	NT *	NT *	NT *	28.00 ± 0.00
Voriconazol (1 µg/disc)	NT *	NT *	NT *	32.50 ± 0.50

* NT—not tested.

**Table 7:** The composition of the studied NADES and HPC-NADES systems.

HPC-NADES Components	HPC-NADES Systems	
	HPC-NADES 17%	HPC-NADES 29%
HPC	1.36 g	1.12 g
ChCl-U 100%	0.73 g choline chloride + 0.63 g urea (1:2 molar ratio)	1.25 g choline chloride + 1.07 g urea (1:2 molar ratio)
ChCl-Gly 100%	0.58 g choline chloride + 0.77 g glycerol (1:2 molar ratio)	1.00 g choline chloride + 1.32 g glycerol (1:2 molar ratio)
ChCl-LA 100%	0.83 g choline chloride + 0.53 g lactic acid (1:1 molar ratio)	1.41 g choline chloride + 0.91 g lactic acid (1:1 molar ratio)
ChCl-CA 100%	0.57 g choline chloride + 0.78 g citric acid (1:1 molar ratio)	0.98 g choline chloride + 1.34 g citric acid (1:1 molar ratio)
water	5.28 g	4.56 g

## Data Availability

Not applicable.

## Supplementary Materials

The following supporting information can be downloaded at: https://www.mdpi.com/article/10.3390/gels8100666/s1, Figure S1. ^1^H NMR spectrum of ChCl-U-H_2_O (1:2:6); Figure S2. 2D ROESY spectrum of ChCl-U-H_2_O (1:2:6 molar ratio); Figure S3. ^1^H NMR spectrum of ChCl-Gly-H_2_O (1:2:11); Figure S4. 2D ROESY spectrum of ChCl-Gly-H_2_O (1:2:11 molar ratio); Figure S5. ^1^H NMR spectrum of ChCl-LA-H_2_O (1:1:7); Figure S6. 2D ROESY spectrum of ChCl-LA-H_2_O (1:1:7 molar ratio); Figure S7. ^1^H NMR spectrum of ChCl-CA-H_2_O (1:1:10); Figure S8. 2D ROESY spectrum of ChCl-CA-H_2_O (1:1:10 molar ratio); Figure S9. 2D ROESY spectrum of HPC-ChCl-U 17%.

## Abbreviation

NADES	Natural Deep Eutectic Solvents	HBD	Hydrogen-Bond Donor
HPC	Hydroxypropyl cellulose	HBA	Hydrogen-Bond Acceptor
LCST	Lower Critical Solution Temperature	HGF	Human Gingival Fibroblast
ChCl	Choline chloride	DSC	Differential Scanning Calorimetry
U	Urea	TGA	Thermogravimetric analysis
CA	Citric acid	DTG	Differential thermogravimetric analysis
LA	Lactic acid	FT-IR	Fourier-Transform Infrared
Gly	Glycerol	NMR	Nuclear Magnetic Resonance
