# Supplementary material for: Biocompatible Self-Assembled Hydrogen-Bonded Gels Based on Natural Deep Eutectic Solvents and Hydroxypropyl Cellulose with Strong Antimicrobial Activity
Source: Gels. 2022 Oct 17;8(10):666. doi: 10.3390/gels8100666 (PMC9601327; doi:10.3390/gels8100666)
Supplement: Supplementary file 1 [file gels-08-00666-s001.zip › gels-1958644-supplementary.pdf]

Supplementary Information

Biocompatible self-assembled hydrogen-bonded gels based on natural deep eutectic solvents and hydroxypropyl cellulose with strong antimicrobial activity

Daniela Filip[1*], Doina Macocinschi[1], Mihaela Balan-Porcarasu[2*], Cristian-Dragos Varganici[3], Raluca-Petronela Dumitriu[1], Dragos Peptanariu[3], Cristina Gabriela Tuchilus[4], Mirela-Fernanda Zaltariov[5*]

[1]*Laboratory of Physical Chemistry of Polymers, "Petru Poni" Institute of Macromolecular Chemistry, Aleea Gr. Ghica Voda 41 A, Iasi, 700487, Romania;*

[2]*Laboratory of Polycondensation and Thermostable Polymers, "Petru Poni" Institute of Macromolecular Chemistry, Aleea Gr. Ghica Voda 41 A, Iasi, 700487, Romania;*

[3]*Centre of Advanced Research in Bionanoconjugates and Biopolymers, "Petru Poni" Institute of Macromolecular Chemistry, Aleea Gr. Ghica Voda 41 A, Iasi, 700487, Romania;*

[4]*"Gr. T. Popa" University of Medicine and Pharmacy, Faculty of Medicine, Microbiology Department, 16 Universitatii Street, 700115, Iasi, Romania*

[5]*Department of Inorganic Polymers, "Petru Poni" Institute of Macromolecular Chemistry, Aleea Gr. Ghica Voda 41 A, Iasi, 700487, Romania;*

Corresponding authors: Mihaela Balan-Porcarasu, e-mail: mihaela.balan@icmpp.ro; Mirela-Fernanda Zaltariov, e-mail: zaltariov.mirela@icmpp.ro

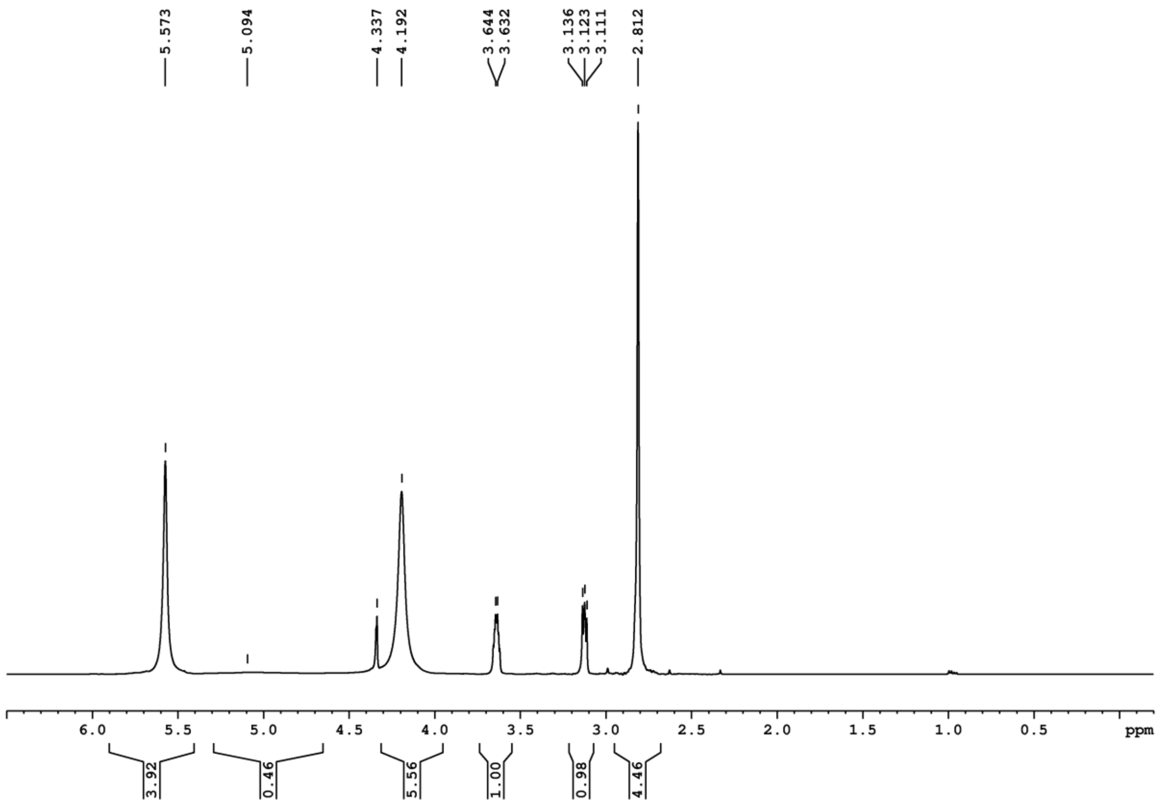

Figure S1. ^{1}H NMR spectrum of ChCl-U-H_2O (1:2:6).

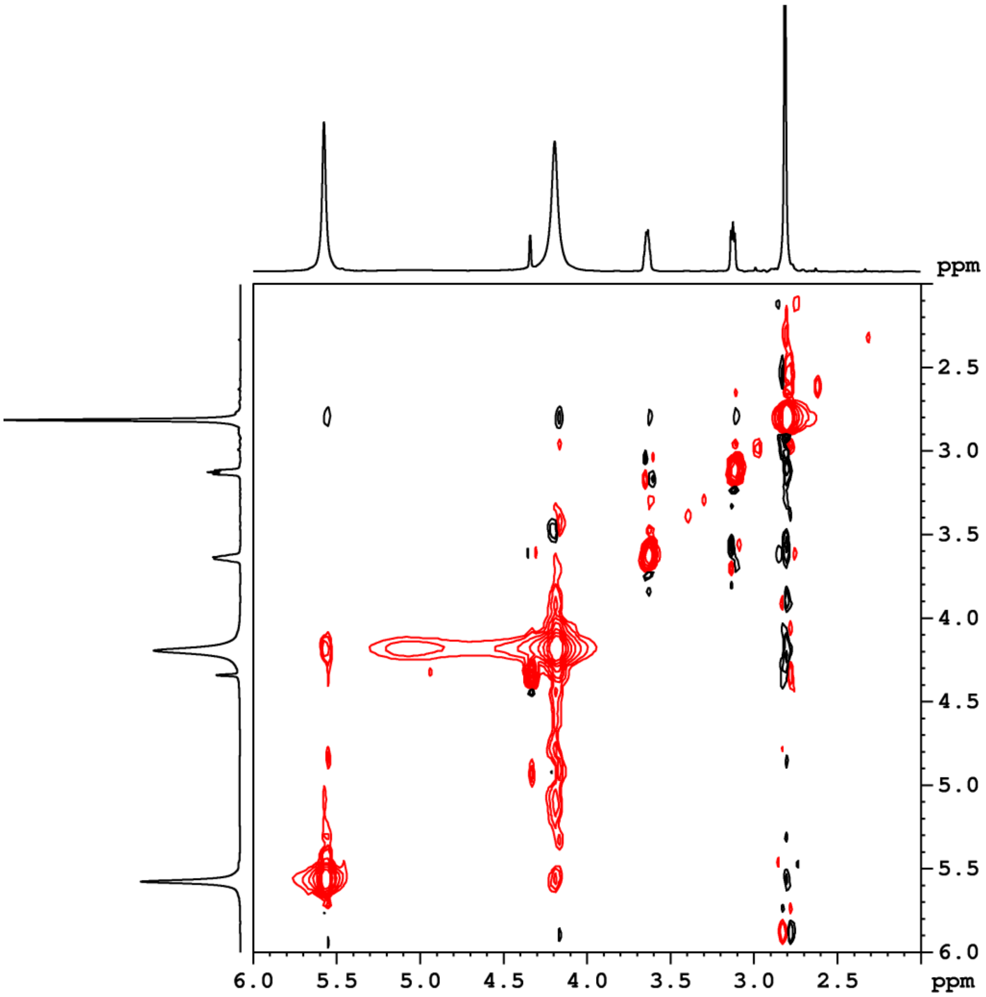

Figure S2. 2D ROESY spectrum of ChCl-U-H_2O (1:2:6 molar ratio).

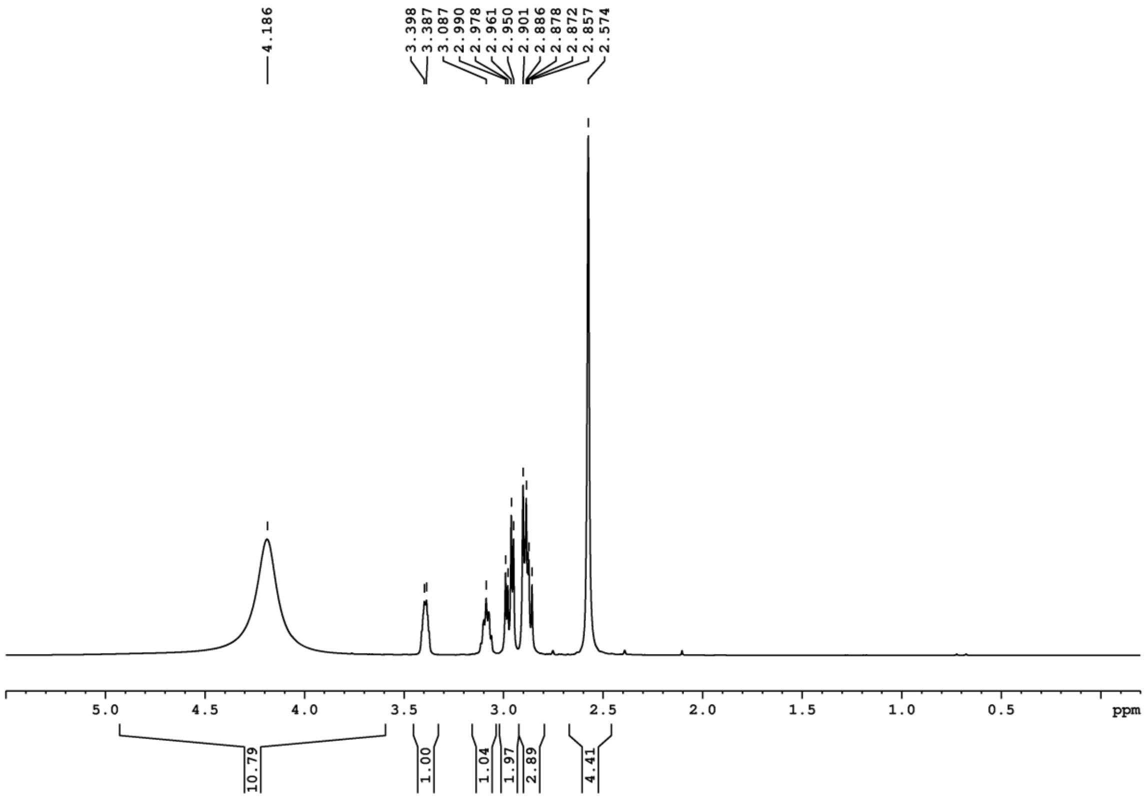

Figure S3. ^{1}H NMR spectrum of ChCl-Gly-H_2O (1:2:11).

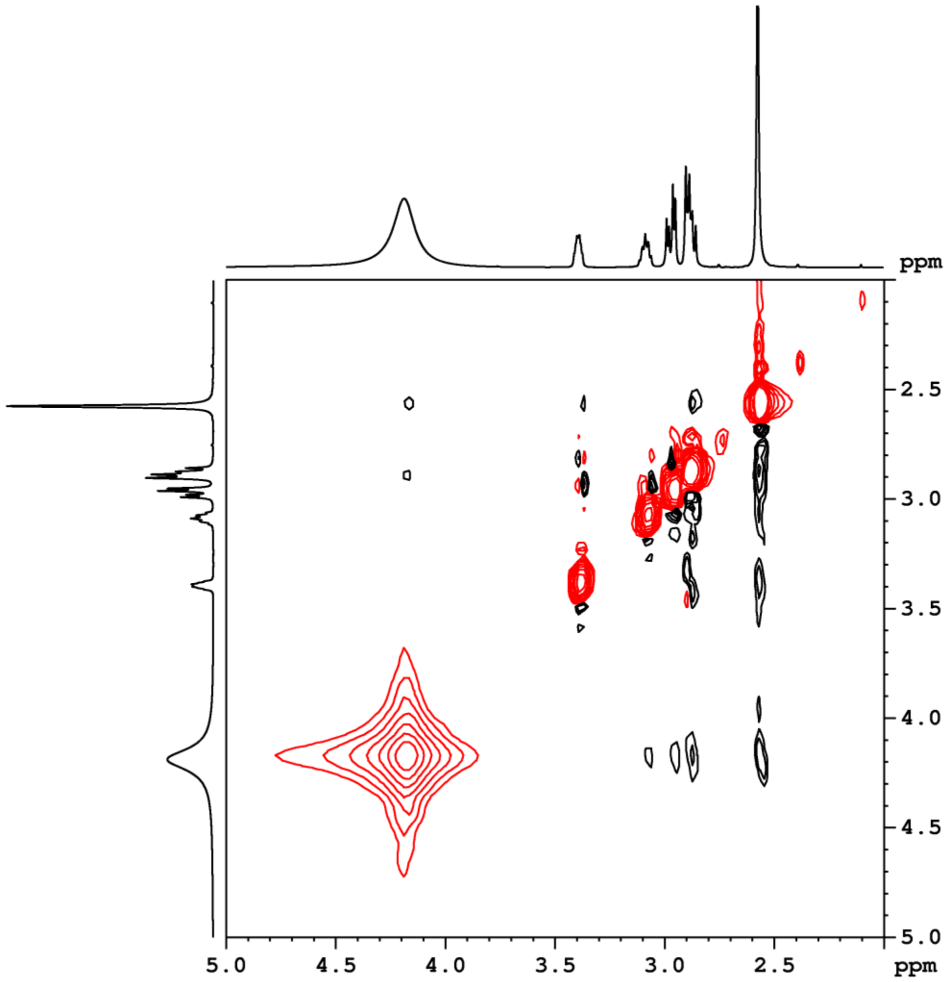

Figure S4. 2D ROESY spectrum of ChCl-Gly-H_2O (1:2:11 molar ratio).

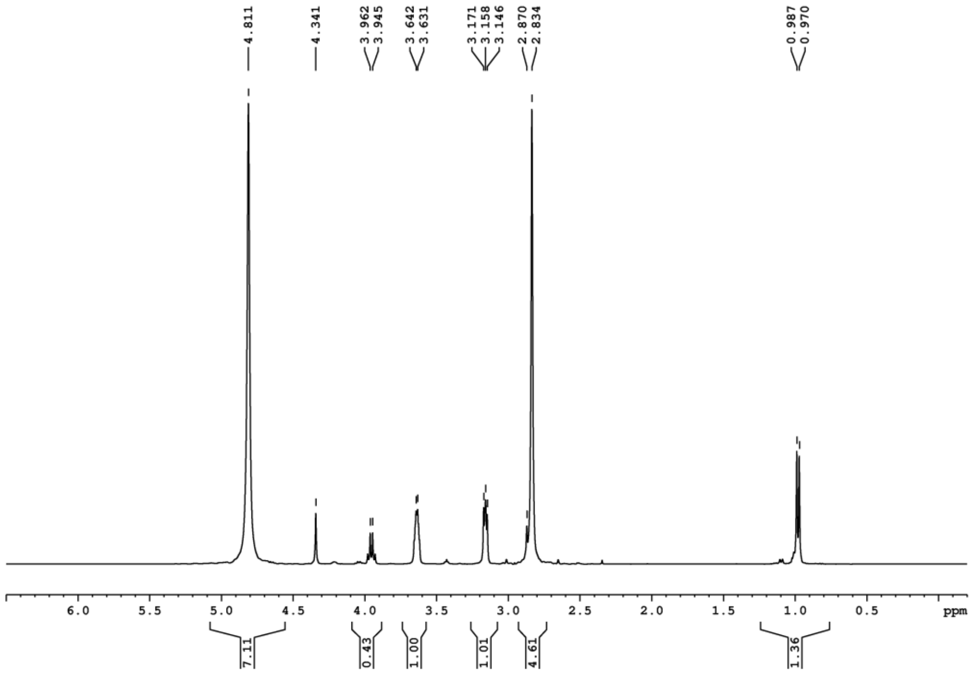

Figure S5. ^{1}H NMR spectrum of ChCl-LA-H_2O (1:1:7).

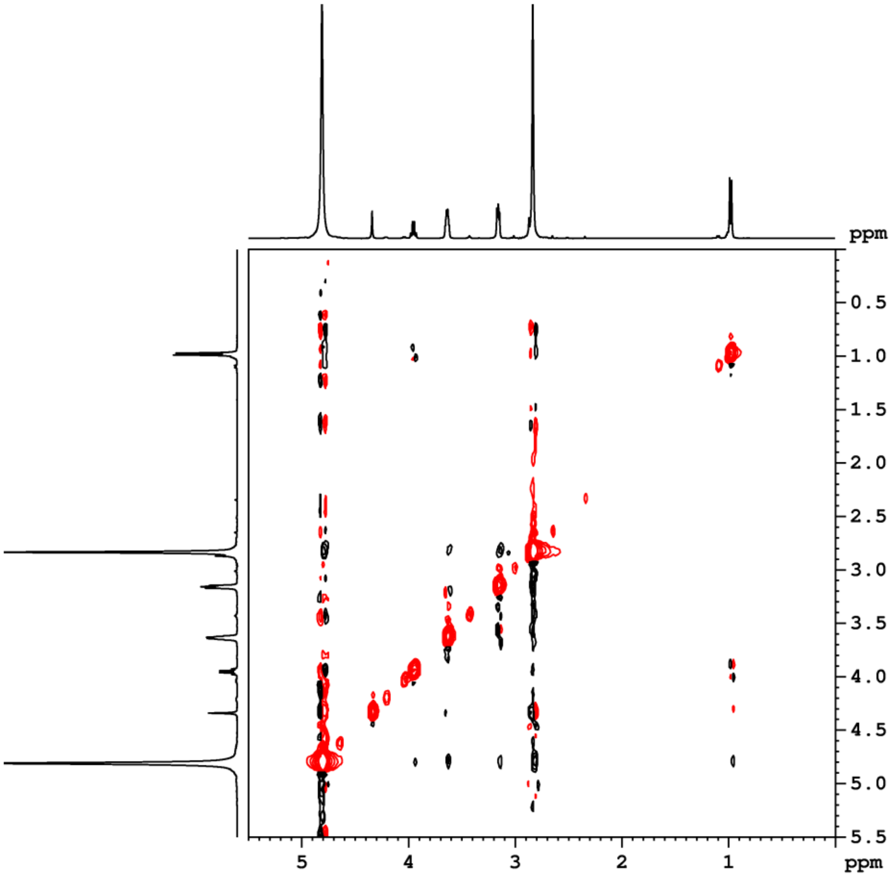

Figure S6. 2D ROESY spectrum of ChCl-LA-H_2O (1:1:7 molar ratio).

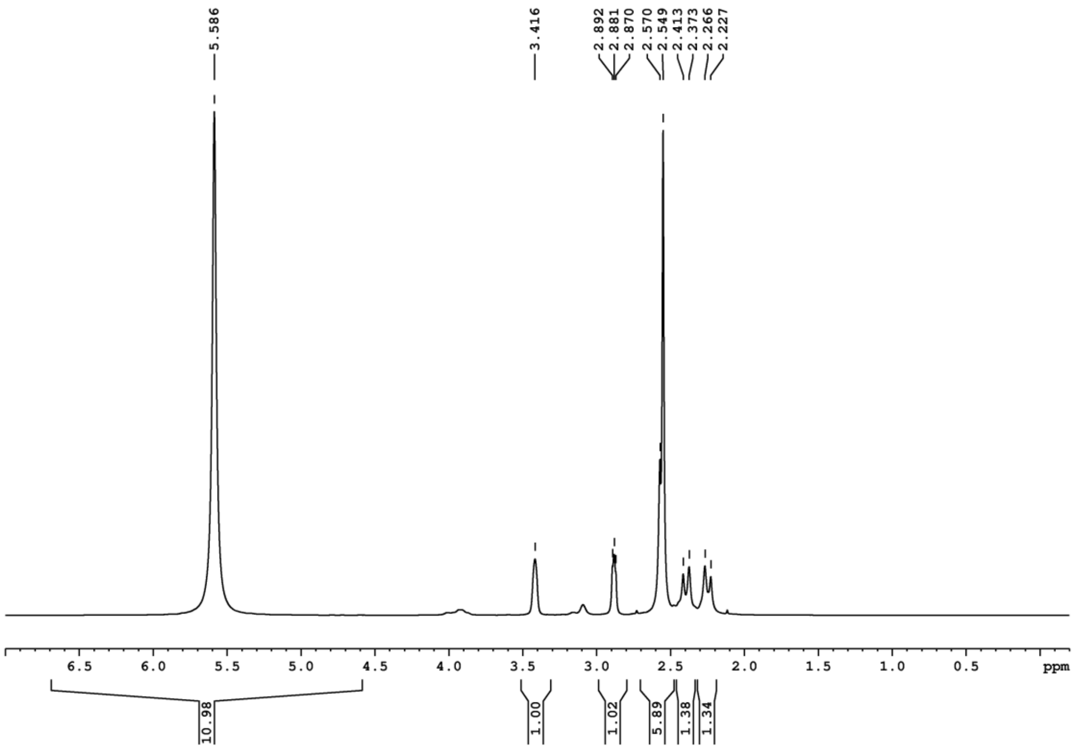

Figure S7. ^{1}H NMR spectrum of ChCl-CA-H_2O (1:1:10).

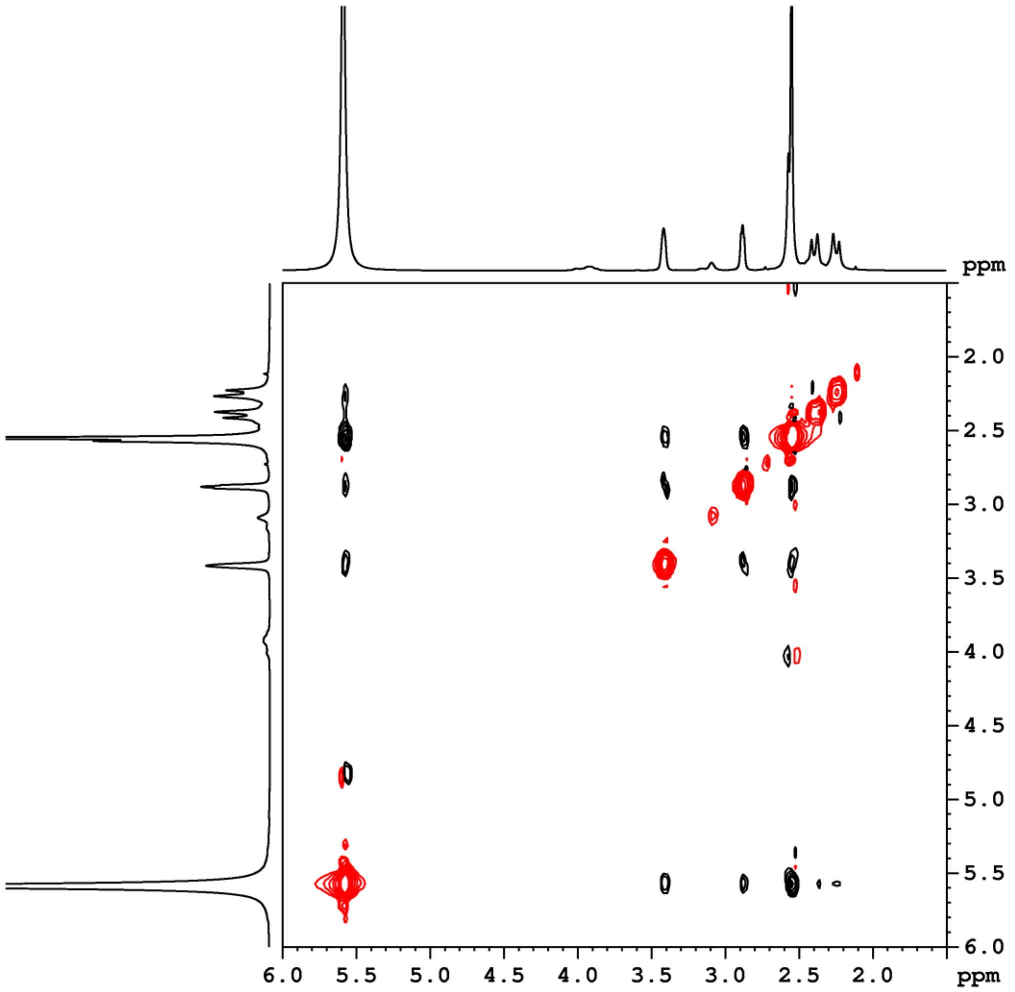

Figure S8. 2D ROESY spectrum of ChCl-CA-H_2O (1:1:10 molar ratio).

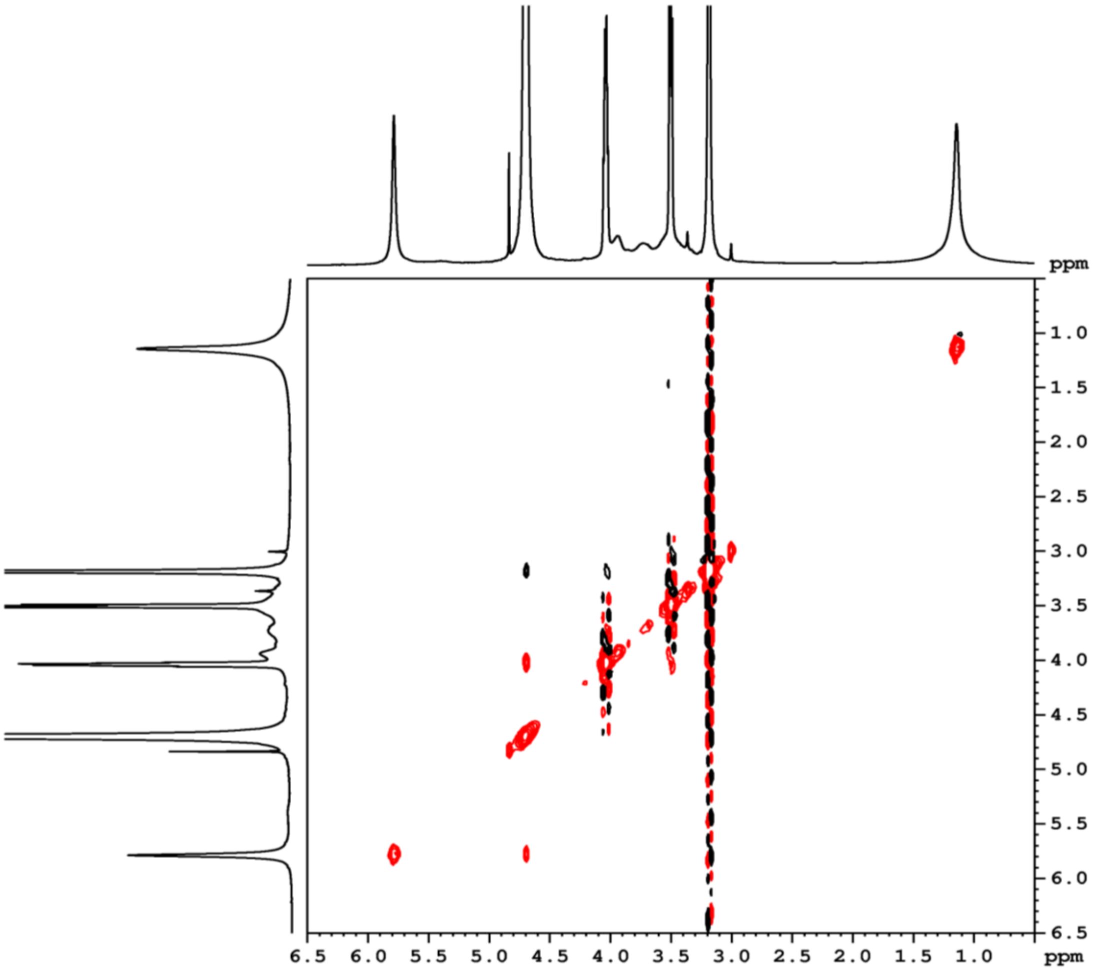

Figure S9. 2D ROESY spectrum of HPC-ChCl-U 17%
